# Supplementary material for: Horizontally acquired papGII-containing pathogenicity islands underlie the emergence of invasive uropathogenic Escherichia coli lineages
Source: Nat Commun. 2020 Nov 24;11:5968. doi: 10.1038/s41467-020-19714-9 (PMC7686366; doi:10.1038/s41467-020-19714-9)
Supplement: Supplementary file 1 — Supplementary Information [file 41467_2020_19714_MOESM1_ESM.pdf]

Supplementary Information

Table of contents

Supplementary Notes 2
Supplementary Figures 4
Supplementary Tables...30
References...45

Supplementary Notes

Supplementary Note 1: Inclusion criteria for isolate collections

The main dataset of this study (385 invasive UPEC isolates, 337 non-invasive UPEC isolates, and 185 fecal isolates giving a total of 907 isolates) comprised genomic data of 14 UPEC isolate collections, 2 fecal isolate collections, and reference strains. Criteria for the inclusion of UPEC isolate collections were the availability of associated metadata on clinical syndromes and/or medical diagnosis, i.e., asymptomatic bacteriuria (ABU), cystitis, pyelonephritis, urinary-source bacteremia, or urosepsis. In addition to isolates sequenced as part of this study, public genomic data of 10 UPEC isolate collections were identified and included.

Asymptomatic bacteriuria (ABU) describes the bacterial colonization of the urinary tract without causing inflammatory responses. The diagnosis of ABU is based on one (in men) or two consecutive (in women) positive urine cultures (typically >100,000 CFU/ml) from individuals without signs or symptoms of urinary tract infection[1]. Cystitis is the inflammation of the bladder or lower urinary tract caused by bacterial infection. The diagnosis of cystitis is based on a positive urine culture (typically >1,000 CFU/ml) from a patient presenting with dysuria, urgency, frequency, pyuria, and/or suprapubic pain. Pyelonephritis is the inflammation of the kidneys or upper urinary tract caused by bacterial infection. The diagnosis of pyelonephritis is based on a positive urine culture (typically >1,000 CFU/ml) from patients presenting with flank pain and/or costovertebral angle tenderness, fever (>38.0 °C), and pyuria, with or without typical symptoms of lower urinary tract infection. Around 30% to 60% of all patients with pyelonephritis develop bacteremia[2–4]: such urinary-source bacteremia is indicated by concurrent positive urine and blood cultures with isolates of the same bacterial species.

To provide phylogenomic context to our analysis, genomic data of two fecal isolate collections (MN_fec, KTE_fec) were included. These originated from healthy volunteers, patients without acute infection, or patients with cystitis (Supplementary Table 1). Fecal isolates from patients with cystitis corresponding to the urinary clone of the same patient (based on whole-genome sequence analyses) were excluded. All fecal isolates were originally used as control strains for genetic comparisons with UPEC isolates. Because the human microbiota presumably presents a reservoir of UPEC, fecal isolates were not considered in genome-wide association studies.

Supplementary Note 2: Distribution of clinical phenotypes and *papGII*+ isolates among phylogroups.

Both UPEC and fecal isolates were predominantly assigned to phylogroup B2 (64% and 41%, respectively; Supplementary Table 2). A majority of the remaining UPEC isolates were part of the D group (15%), whereas most remaining fecal isolates were part of phylogroups A (22%), B1 (15%), or D (15%). Phylogroup C consisted exclusively of UPEC isolates. Clustering of fecal isolates in sublineages of phylogroups A and B1 hinted toward the existence of a truly commensal *E. coli* type not able to colonize the urinary tract (Fig. 1). Notably, the phylogeny of groups A and B1 presented a deep branching population structure. In contrast, most isolates in phylogroups B2 and D grouped into few lineages of highly related isolates, in line with a previously reported expansion of a few successful pandemic lineages[5]. Invasive UPEC isolates were more commonly associated with phylogroups B2 and D (86%) than non-invasive UPEC isolates (71%). Almost all (96%) *papGII*+ isolates were part of phylogroups B2, D, or F.

Supplementary Note 3: Distribution of iron uptake systems

Seven of the 22 investigated iron uptake systems were significantly more prevalent in invasive compared to non-invasive UPEC isolates (Supplementary Table 10a). In addition to the *iuc* locus, which reached pan-genome wide significance, these included *sit*, *chu*, *cjr*, *ireA*, *iha*, and *fyu/ybt*. Among

isolates of phylogroup B2, *iuc*, *iha*, and *ireA* were significantly more prevalent in invasive than in non-invasive UPEC isolates (Supplementary Table 10b). The yersiniabactin gene cluster *fyu/ybt*, encoded by the high-pathogenicity island HPI, and the heme uptake system *chu*, which are both considered to play a major role in extra-intestinal virulence[6,7], were present in >90% of all isolates in phylogroups B2, C, F or phylogroups B2, D, F, G respectively (Supplementary Table 10c).

Supplementary Figures

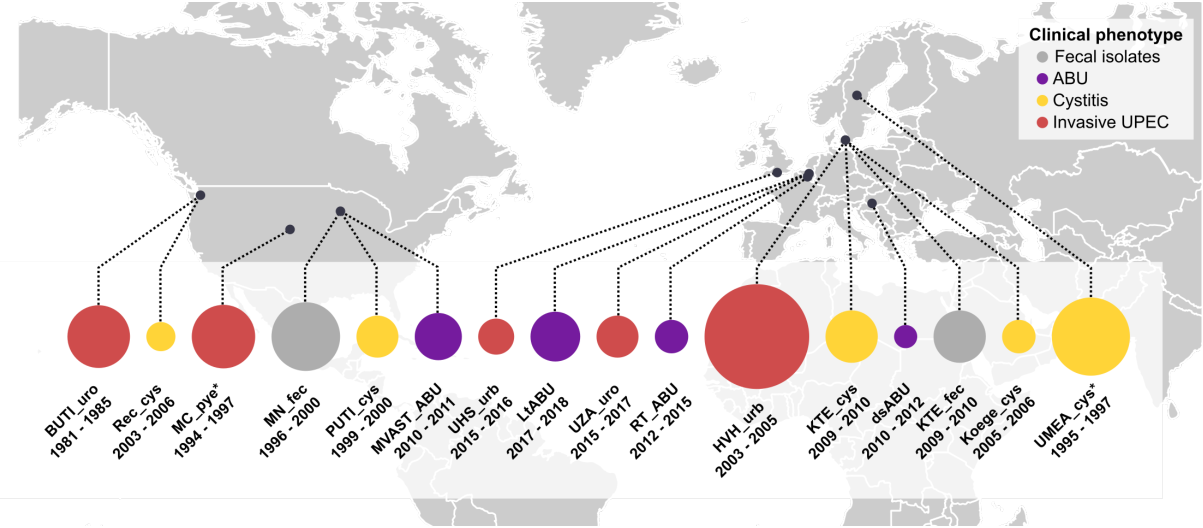

Supplementary Fig. 1. Geographical origin of *E. coli* collections included in the main dataset of this study.

Clinical phenotypes and the number of isolates in each collection are illustrated by different colors and scales of the circles. The isolation time span of each collection is annotated. Asterisks indicate multicenter studies. The map was created in R using the maps package[8]. ABU: asymptomatic bacteriuria, UPEC: uropathogenic *E. coli*.

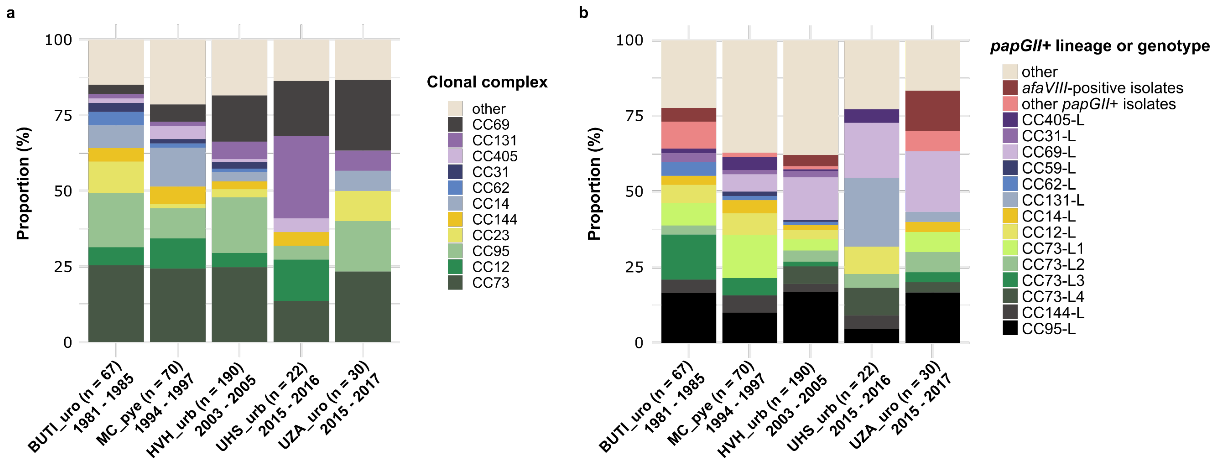

Supplementary Fig. 2. Proportion of dominant lineages in the five collections consisting of invasive uropathogenic *E. coli* isolates.

These include collections of *E. coli* isolates associated with pyelonephritis (MC_pye) and urinary-source bacteremia (HVH_urb, UHS_urb, BUTI_uro, UZA_uro). The number of isolates and isolation time span of each collection is annotated. (a) Proportion of the dominant clonal complexes (CC). The eleven dominant CCs accounted for 79% to 87% of isolates in each of the five collections. More recent collections consisted of a higher proportion of CC131 and CC69 isolates. (b) Proportion of *papGII*+ lineages. The proportion of *papGII*+ isolates that were not part of the 14 identified *papGII*+ lineages as well as the proportion of *afaVIII*-positive isolates are also indicated. One isolate (U6_BUTI_Uro, CC23, collection BUTI_uro, not part of a *papGII*+ lineage) carried both *papGII* and *afaVIII* and was assigned to "other *papGII*+ isolates". Source data are provided in Supplementary Data 1.

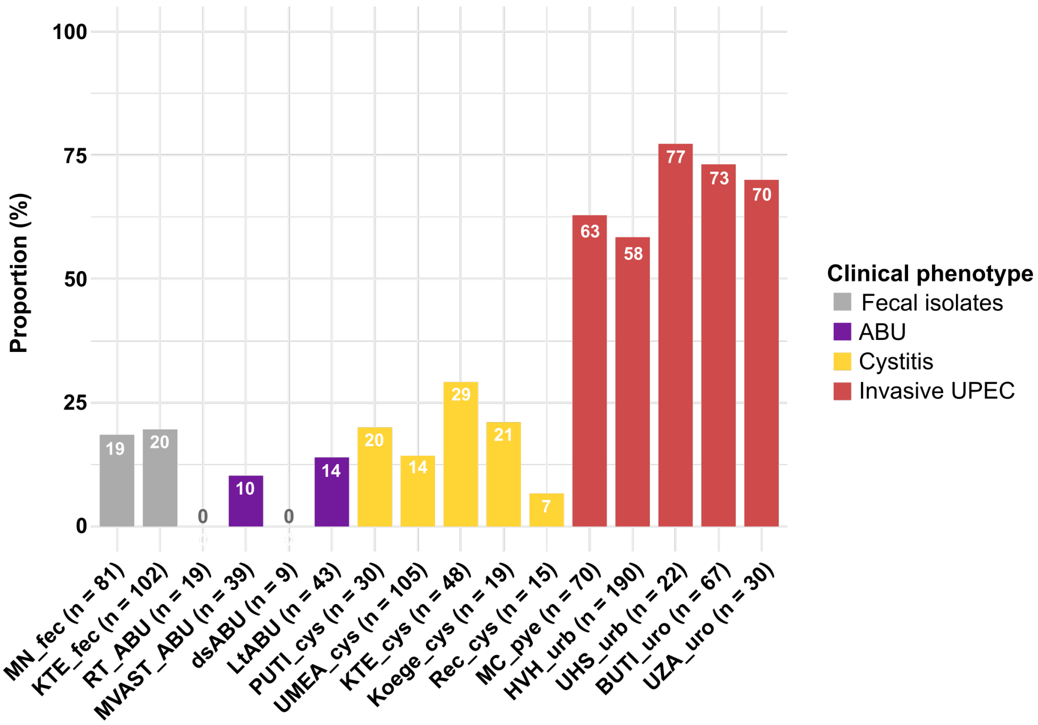

Supplementary Fig. 3. Proportion of *papGII*+ isolates in each *E. coli* collection.

The clinical phenotype of each collection is indicated. Source data are provided in Supplementary Data 1. ABU: asymptomatic bacteriuria, UPEC: uropathogenic *E. coli*

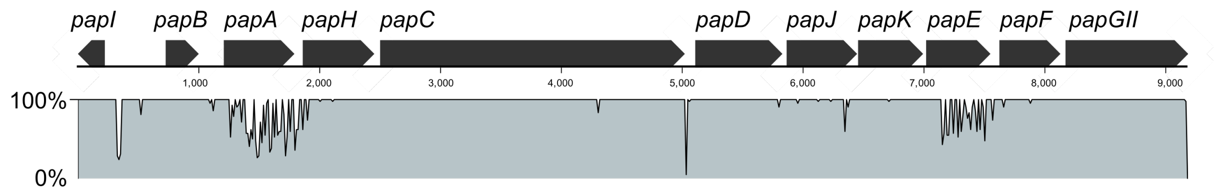

Supplementary Fig. 4. Genetic organization and nucleotide sequence conservation of the *pap* operon.

The plot indicates nucleotide sequence conservation per site based on an alignment of 42 *papGII* operons from resolved pathogenicity islands. The operons were aligned using Muscle[9] and the sequence conservation calculated and visualized in CLC Sequence Viewer 8.0.

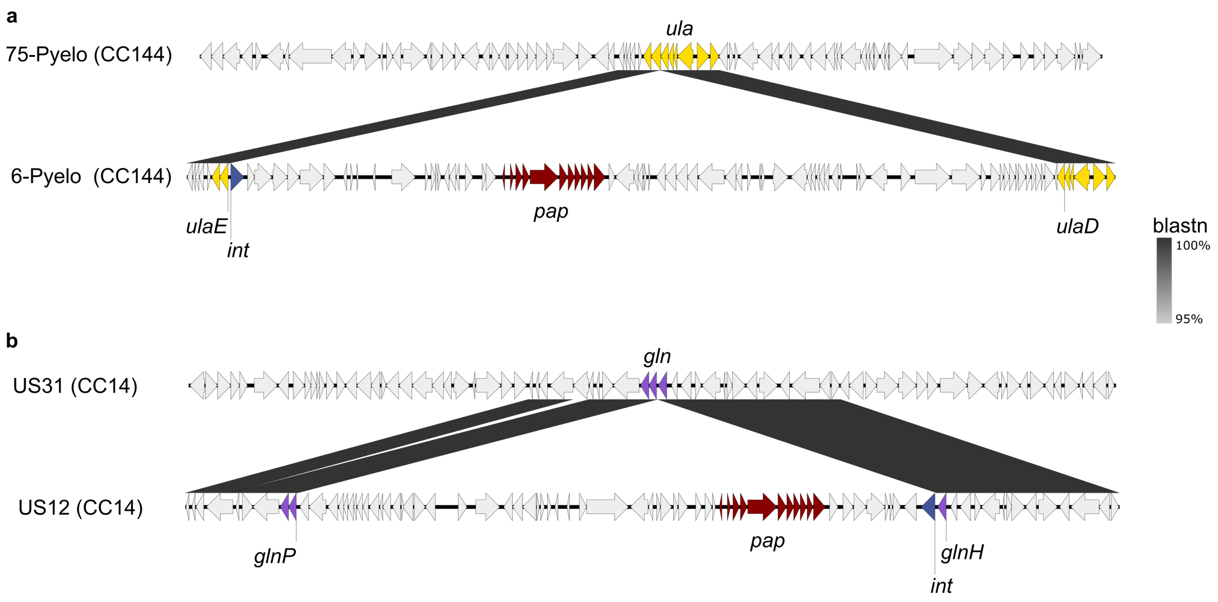

Supplementary Fig. 5. Genetic context of two novel pathogenicity island (PAI) integration sites *ula* and *gln*.

(a) Comparison of the region surrounding the *papGII*-containing PAI of isolate 6-Pyelo (CC144) with the chromosomal backbone of isolate 75-Pyelo (CC144), which lacked insertions at the *ula* site. $PAI_{6\text{-}Pyelo\text{-}ula}$ (Type I *papGII*+ PAI) was integrated between the genes *ulaE* and *ulaD*. The *ulaRGABCDEF* operon (yellow) encodes an L-ascorbate phosphotransferase transport system. (b) Comparison of the region surrounding the *papGII*-containing PAI of isolate US12 (CC14) with the chromosomal backbone of isolate US31 (CC14), which lacked insertions at the *gln* site. $PAI_{US12\text{-}gln}$ (Type IV *papGII*+ PAI) was integrated between *glnP* and *glnH* of the *glnHPQ* operon (purple), which encodes an ABC glutamine transport system. Integrase genes (*int*) and the *pap* operon are marked. The gradient scale shows the level of nucleotide identity. Sequence comparisons were performed using EasyFig[10].

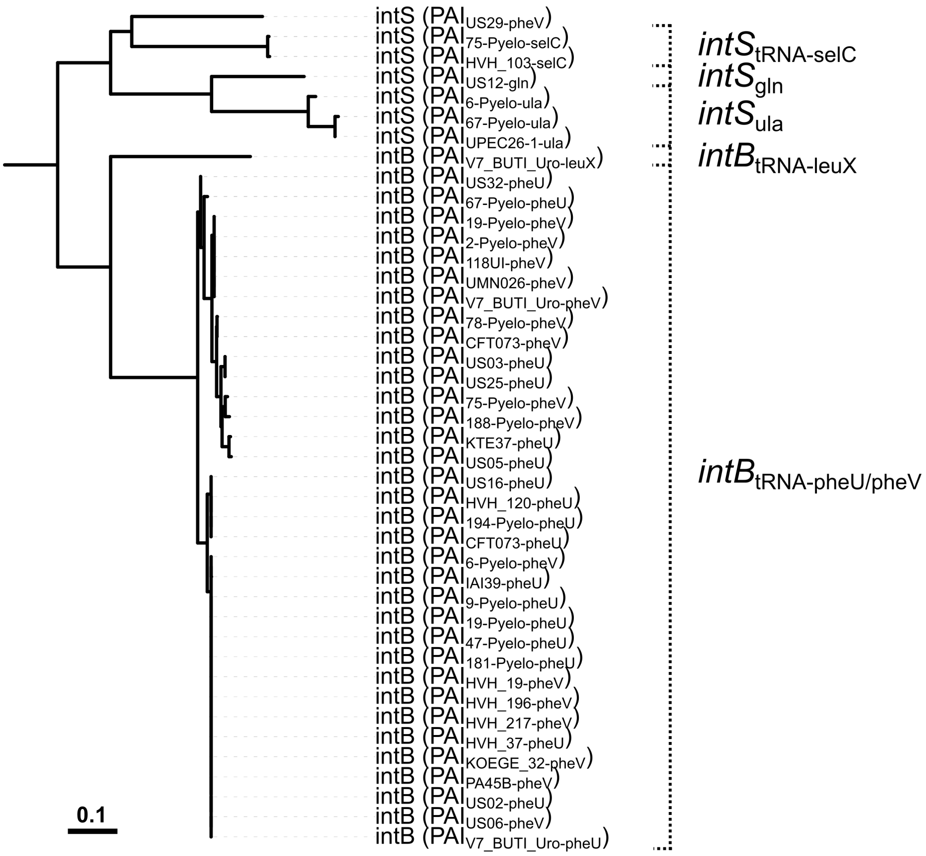

Supplementary Fig. 6. Midpoint-rooted maximum-likelihood tree for *intS* and *intB* integrase genes identified on 42 resolved *papGII*-containing pathogenicity islands (PAIs).

Integrase genes were aligned using Muscle and the phylogeny reconstructed in Mega-X using a GTR model. DNA sequences of the integrase genes clustered according to their integration site. Integrase genes identified at the tRNA-*pheU* and tRNA-*pheV* sites fell in the same cluster, often sharing an identical sequence. An exception was the integrase gene found in PAI$_{\text{US29-pheV}}$.

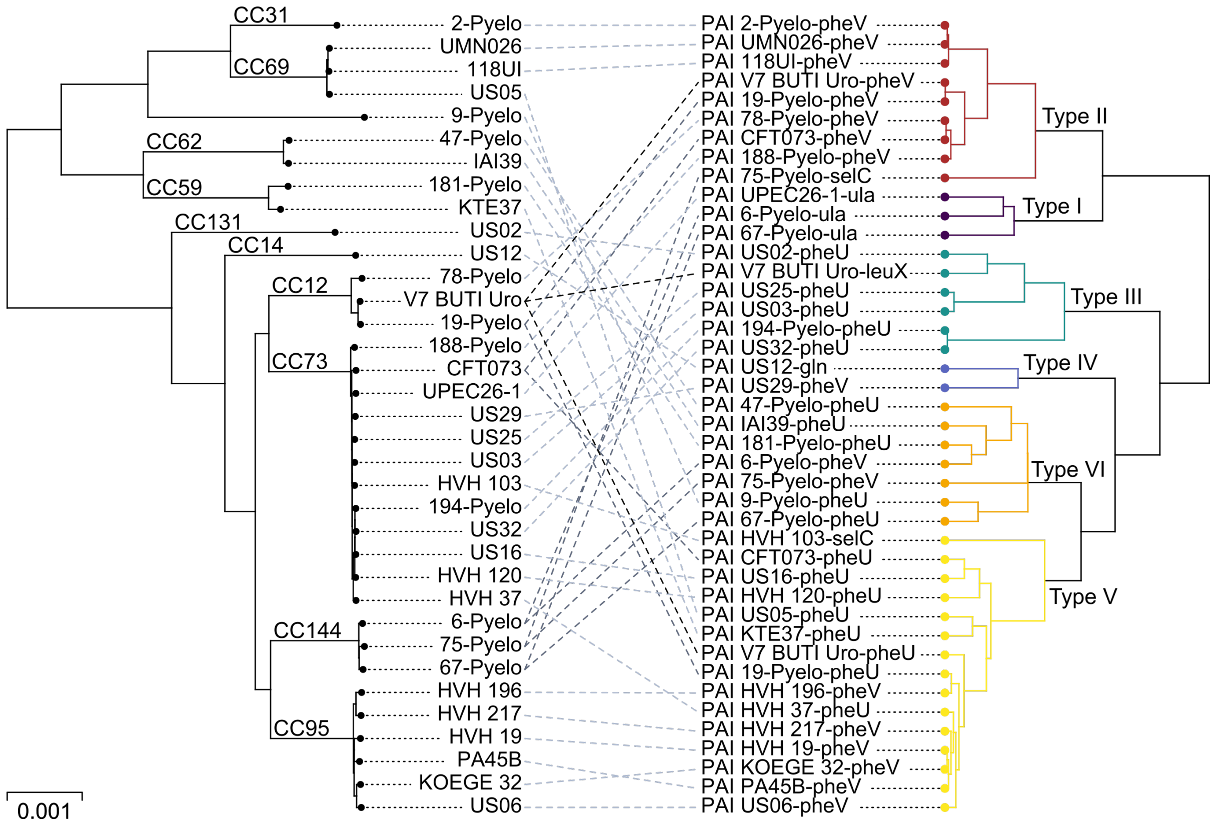

Supplementary Fig. 7. Tanglegram depicting the core genome phylogeny of 35 representative *papGII*+ isolates and the similarity of 42 *papGII*+ pathogenicity islands (PAIs) identified in their genomes.

Left: midpoint-rooted maximum-likelihood phylogeny of 35 high-quality genome assemblies from *papGII*+ isolates based on a separate 3.412 Mbp core genome alignment (reference genome CFT073, GCA_000007445.1). Clonal complexes (CCs) are annotated. The scale bar indicates the number of substitutions per site. Right: sequence similarity of 42 resolved *papGII*+ PAIs identified in the 35 assemblies based on pairwise mash distance. Six isolates (V7_BUTI_Uro, 19-Pyelo, CFT073, 6-Pyelo, 75-Pyelo, 67-Pyelo) contained more than one *papGII*+ PAI. *papGII*+ PAI types are annotated. Dotted lines connect PAIs with the position of the associated isolate in the phylogeny. The tanglegram was created using the R package phylools[11].

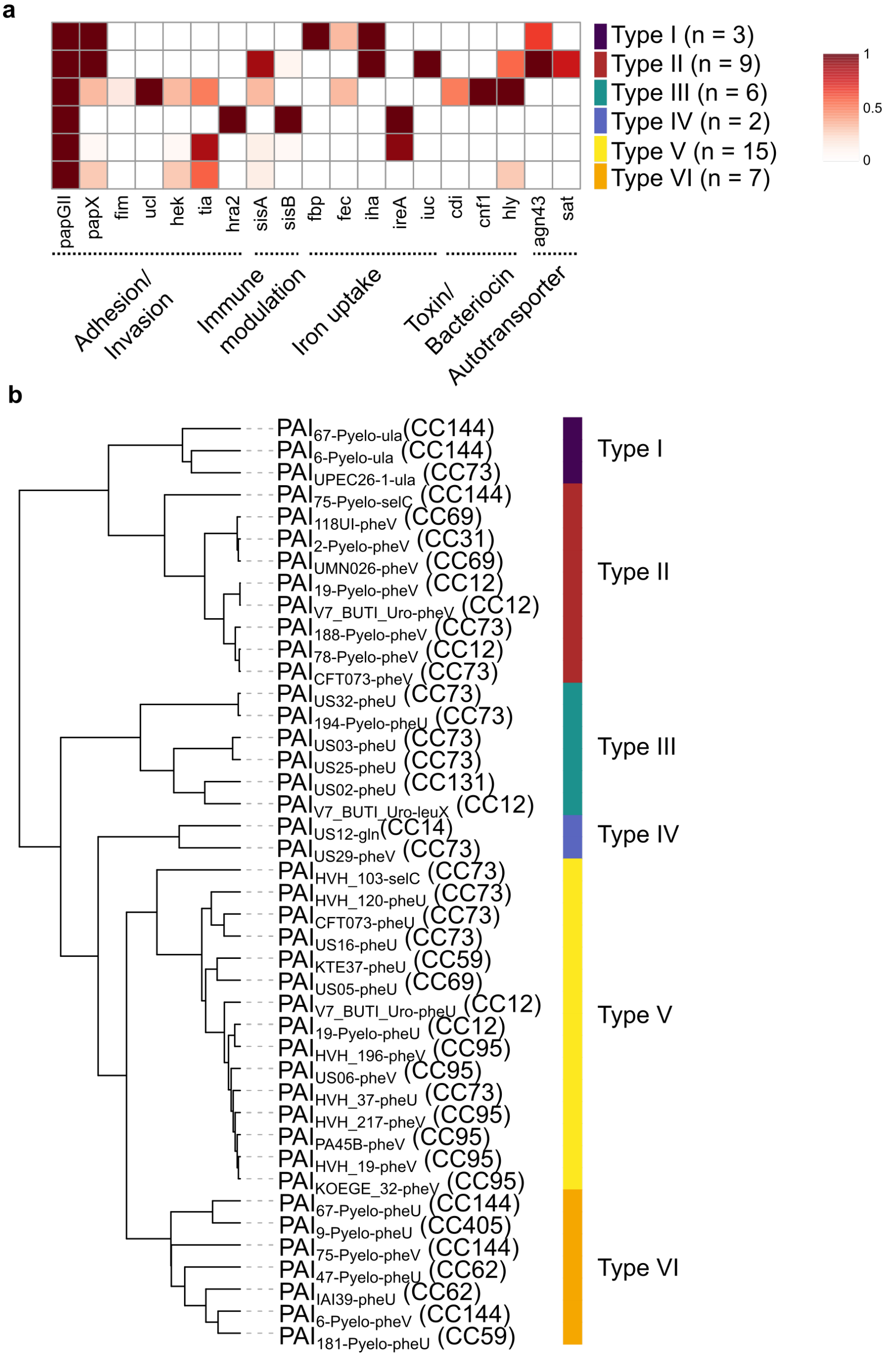

Supplementary Fig. 8. Comparison of pathogenicity islands (PAIs) containing the *papGII* locus (*papGII*+ PAIs).

(a) Heatmap showing the frequency of virulence-associated genes in the six identified *papGII*+ PAI types based on their presence on 42 resolved PAIs. (b) Similarity of 42 fully resolved *papGII*+ PAIs identified in 35 high-quality genome assemblies based on pairwise mash distances, an alignment-free estimation of the sequence similarity relying on k-mer based scoring. PAIs grouped into six types by applying a mash distance cutoff of 0.04. The clonal complex (CC) of the isolate harboring the PAI is annotated.

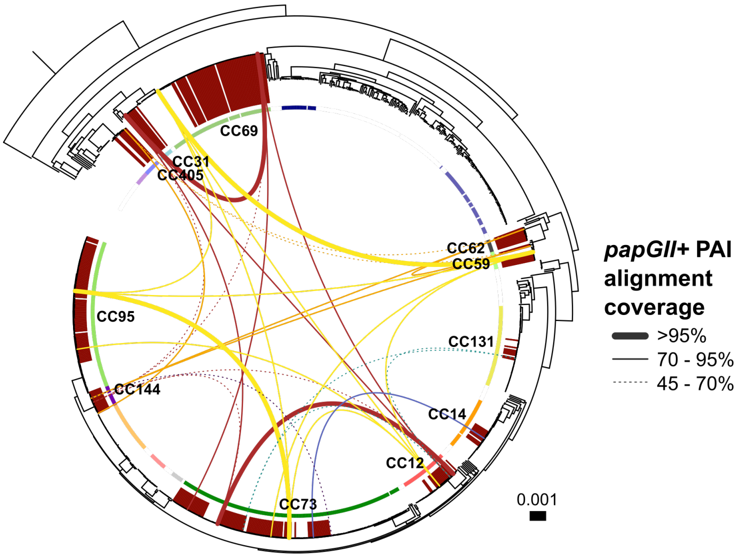

Supplementary Fig. 9. Similarity of *papGII*+ pathogenicity islands (PAIs) across different clonal complexes visualized on the inverted core genome phylogeny as shown in Fig. 1.

Isolates of different clonal complexes carrying resolved PAIs of the same *papGII*+ PAI type are connected with lines colored according to the *papGII*+ PAI type (Supplementary Fig. 8b). Line width and style indicate the alignment coverage of the most similar (based on alignment coverage) *papGII*+ PAIs in isolates of respective clonal complexes. High similarities indicate potential horizontal transfer events of *papGII*+ PAIs. Isolates in *papGII*+ lineages (red shades) and predominant clonal complexes (inner ring) are shown. The tree was visualized using iTOL[12].

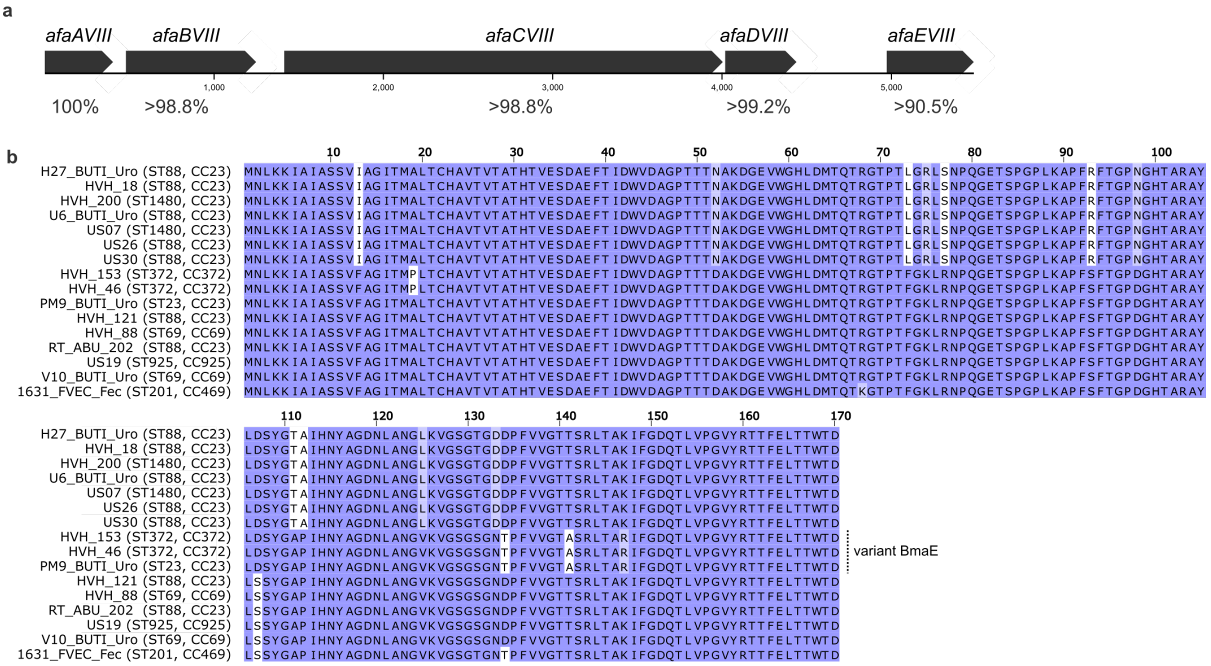

Supplementary Fig. 10. Genetic organization of *afaVIII* operons and conservation of AfaEVIII protein sequences.

(a) Genetic organization of *afaVIII* operons. *afaVIII* operon assemblies were resolved in 17 out of 18 *afaVIII*+ isolates. In 16/17 isolates, the operon was complete; in one isolate (RT_ABU_54), the operon was disrupted by an IS element inserted between *afaDVIII* and *afaEVIII*. Percentages indicate the amino acid sequence identities of each predicted gene product from the 16 resolved and complete *afaVIII* operon assemblies. AfaAVIII, AfaBVIII, AfaCVIII, and AfaDVIII protein sequences were conserved, whereas AfaEVIII occurred in different variants. (b) Multiple sequence alignment of 16 AfaEVIII predicted protein sequences from complete and resolved *afaEVIII* operon assemblies. Three sequences corresponded to an AfaVIII-variant previously described as BmaE (M-agglutinin)[13]. Sequence types (ST) and clonal complexes (CC) are annotated. The alignment was performed using Muscle[9] and visualized in JalView[14].

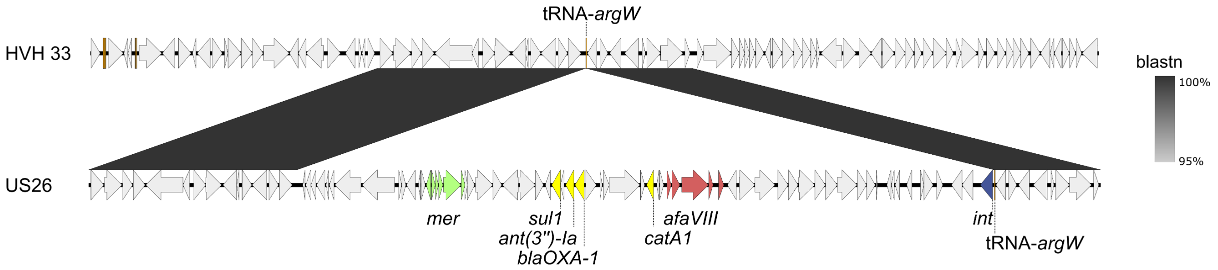

Supplementary Fig. 11. Genetic context of an *afaVIII*-containing pathogenicity island.

The pathogenicity-resistance hybrid island $PAI_{US26\text{-}argW}$ of isolate US26 (ST88, CC23) was inserted at the tRNA-*argW* site. The island contained the operon *afaVIII* (colored in red) as well as multiple antibiotic resistance genes and the mercury resistance operon *merRTPCAD*. The tRNA genes, integrase genes, and the virulence-associated gene *agn43* encoding Antigen 43 are labeled. The gradient scale shows the level of nucleotide identity. Sequence comparisons were performed using EasyFig[10].

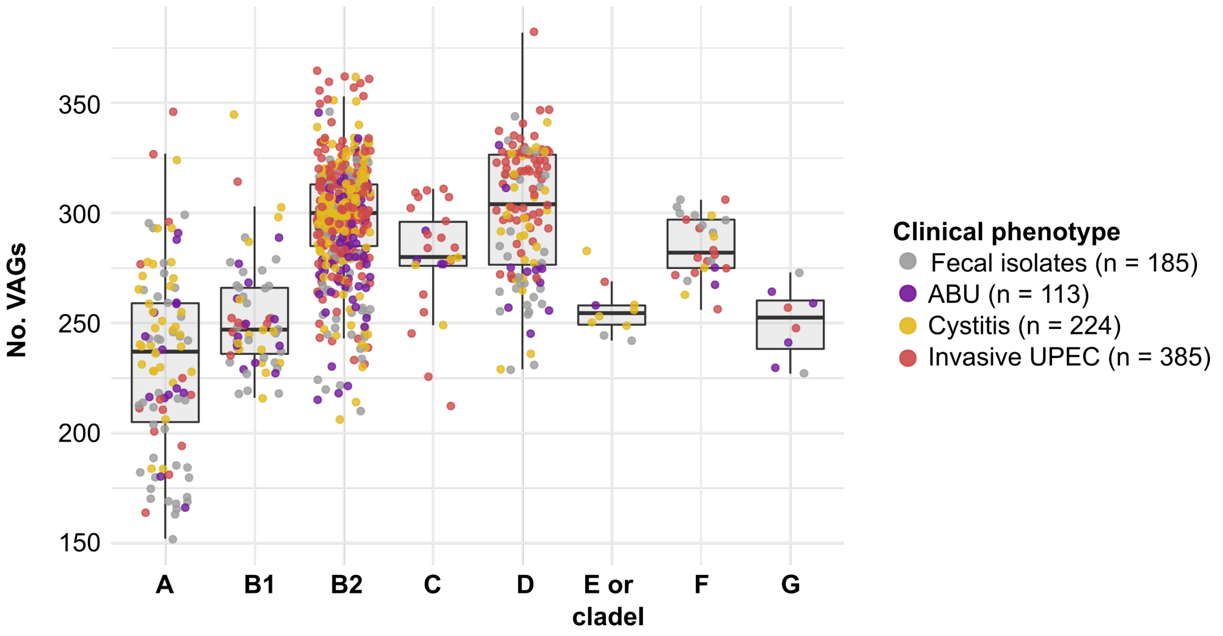

Exact *P* values

	A (n = 99)	B1 (n = 63)	B2 (n = 541)	C (n = 25)	D (n = 135)	E or clade I (n = 10)	F (n = 26)
B1 (n = 63)	**3.62E-02**						
B2 (n = 541)	**4.24E-37**	**5.10E-23**					
C (n = 25)	**8.00E-06**	**6.16E-04**	**9.85E-03**				
D (n = 135)	**3.48E-25**	**1.37E-17**	1.00E+00	**1.82E-02**			
E or clade I (n = 10)	9.75E-01	1.00E+00	**1.96E-04**	1.72E-01	**3.23E-04**		
F (n = 26)	**3.49E-08**	**5.41E-07**	**7.38E-03**	1.00E+00	5.10E-02	**2.83E-03**	
G (n = 8)	1.00E+00	1.00E+00	**6.85E-04**	1.12E-01	**1.25E-03**	1.00E+00	**3.75E-03**

Supplementary Fig. 12. Number of virulence-associated genes (VAGs) per isolate by clinical phenotype and phylogroup.

Boxplots showing the number of VAGs per isolate by phylogroup. Clinical phenotypes of each isolate are indicated in different colors. Average numbers are provided in Supplementary Table 7. Source data are provided in Supplementary Data 8. Exact *P* values for pairwise comparisons between phylogroups (two-side Mann-Whitney U test, Bonferroni corrected) are given in the table. Boxplot center lines: median; box limits: upper and lower quartiles; whiskers extend from the hinges to the highest and lowest values that are within 1.5×IQR of the hinges. ABU: asymptomatic bacteriuria, UPEC: uropathogenic *E. coli*

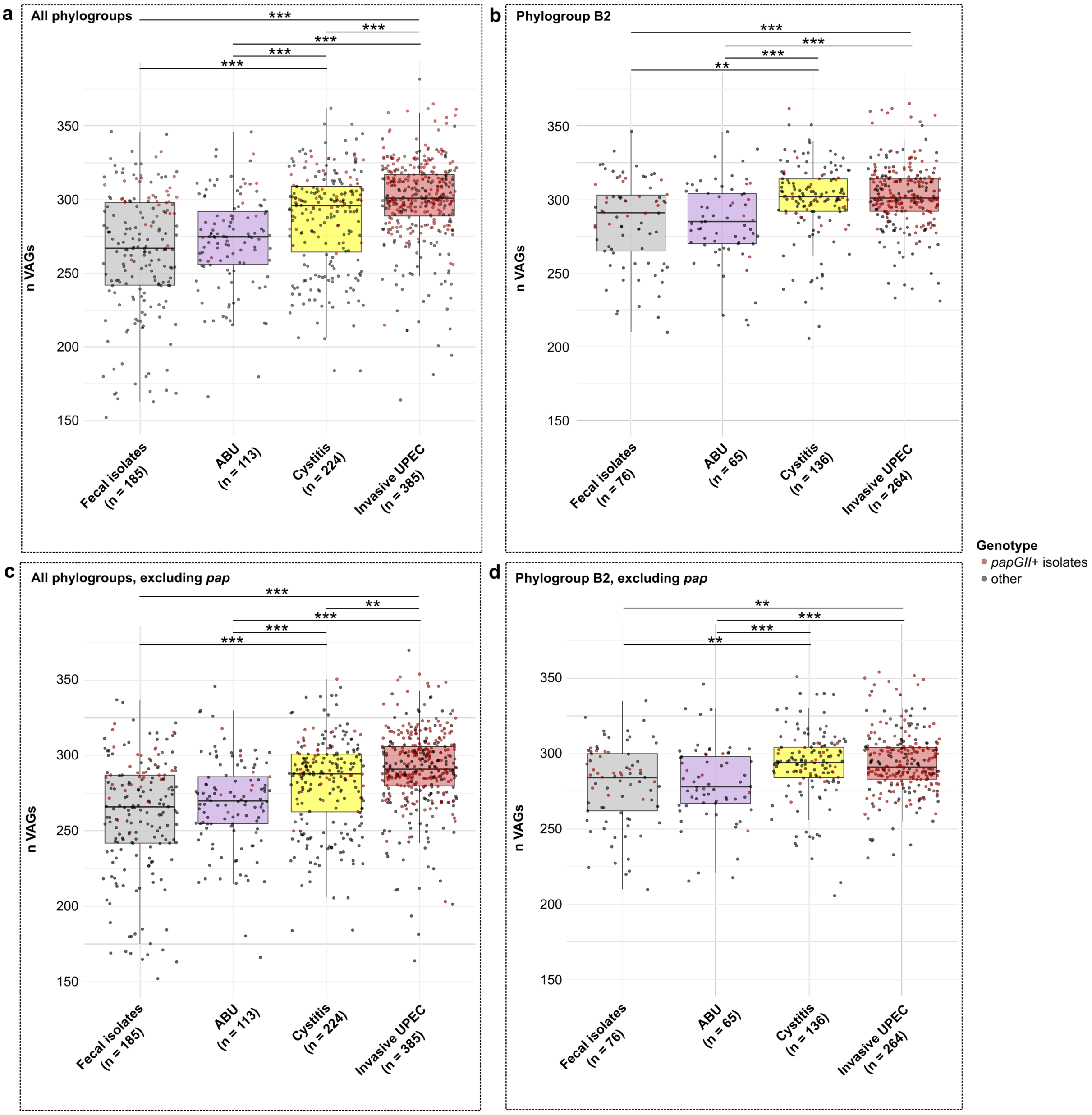

Exact *P* values

	All phylogroups			Phylogroup B2			All phylogroups, excluding *pap*			Phylogroup B2, excluding *pap*		
	ABU	Cystitis	Fecal	ABU	Cystitis	Fecal	ABU	Cystitis	Fecal	ABU	Cystitis	Fecal
Cystitis	9.51E-05			2.75E-04			1.51E-04			7.46E-04		
Fecal	1.00E+00	7.64E-08		1.00E+00	2.63E-03		1.00E+00	1.18E-07		1.00E+00	4.73E-03	
invasive UPEC	5.49E-17	8.22E-05	1.34E-23	1.38E-06	1.00E+00	1.31E-04	6.55E-14	2.77E-03	1.12E-20	7.49E-05	1.00E+00	1.26E-03

Supplementary Fig. 13. Number of virulence-associated genes (VAGs) in *E. coli* isolates associated with asymptomatic bacteriuria (ABU), cystitis, invasive UTI, or isolated from feces.

(a) Boxplot diagram showing the number of VAGs in isolates per clinical phenotype in the complete dataset and (b) in phylogroup B2. (c) Number of VAGs in isolates per clinical phenotype excluding all *pap* genes in the complete dataset and (d) in phylogroup B2. *papGII*+ isolates are highlighted in red. Asterisks indicate *P* values for significant differences between groups (***P* < 0.01, ****P* < 0.001, two-sided Mann-Whitney U test, Bonferroni corrected). Exact *P* values are provided in the table. The average number of VAGs per isolate classified by virulence factor class is given in Supplementary Table 6. Source data are provided in Supplementary Data 8. Boxplot center lines: median; box limits: upper and lower quartiles; whiskers extend from the hinges to the highest and lowest values that are within 1.5×IQR of the hinges.

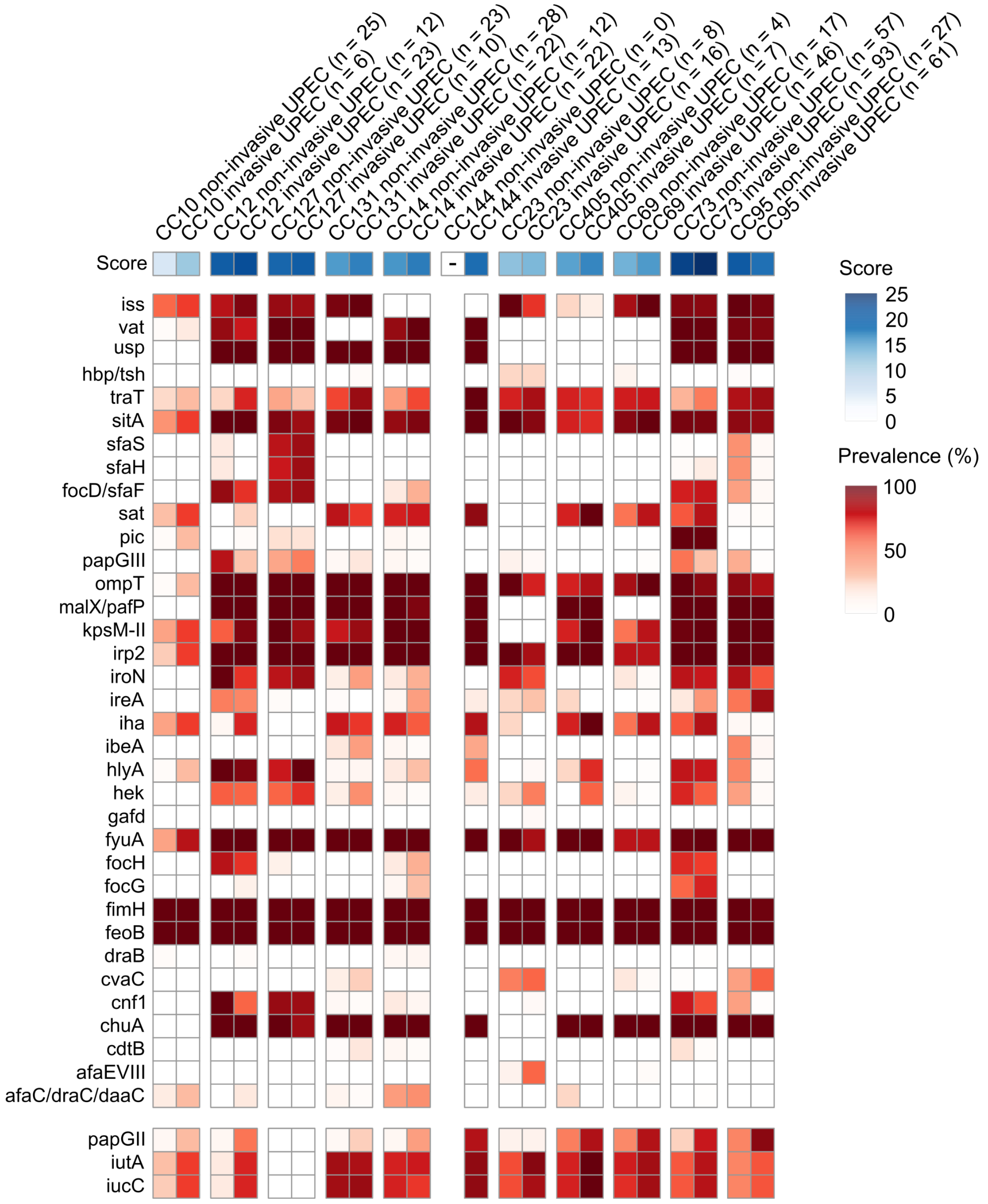

Supplementary Fig. 14. Distribution of putative urovirulence factors by clonal complex (CC) and clinical phenotype.

Heatmap showing the prevalence of selected VAGs described in previous studies[7,15–17] as putative urovirulence factors among common CCs (>10 isolates) according to the scale bar. The score indicates the average number of putative urovirulence factors per isolate. On a genome-wide level, only *papGII* and *iuc* (shown at the bottom) were significantly associated with invasive UPEC (Fig. 2).

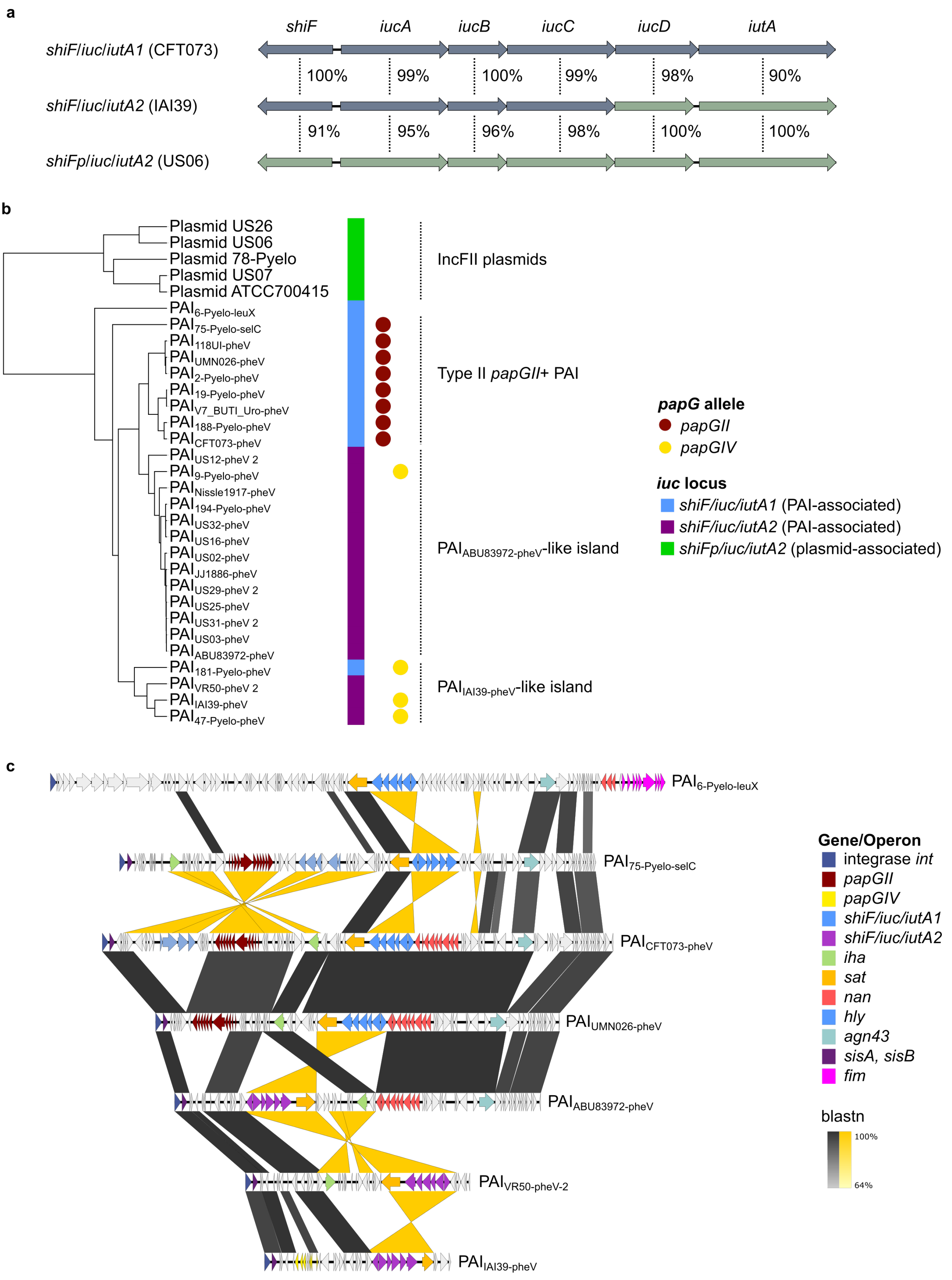

Supplementary Fig. 15. Aerobactin loci *iuc* and associated mobile genetic elements.

(a) Genetic organization and sequence comparison of three *iuc* loci variants identified in isolates CFT073, IAI39 and US06. Numbers indicate amino acid sequence identities. Genes with >98% amino acid sequence identities are labeled in the same color. The three variants were found to be highly conserved in the investigated genomic dataset of 907 isolates, with almost all (97%) identified *iuc* loci

sharing >99% nucleotide sequence coverage and >99% nucleotide sequence identity to either of the three variants. BLAST hits with sequence coverage of <99% were often due to incomplete assemblies. (b) Clustering of resolved mobile genetic elements (MGEs) containing the *iuc* locus as identified in complete or near-complete genome assemblies by genetic similarity. MGEs were hierarchically clustered based on pairwise mash distance. The identified *iuc* variant and the presence of *papG* variants on the MGEs are indicated. *shiFp/iuc/iutA2* was identified on plasmids, while *shiF/iuc/iutA1* and *shiF/iuc/iutA2* were identified on pathogenicity islands (PAIs). PAIs with the *papGIV* locus only contained remnants of the *papGIV* operon. Four islands ($PAI_{US12\text{-}pheV\text{-}2}$, $PAI_{US29\text{-}pheV\text{-}2}$, $PAI_{US31\text{-}pheV\text{-}2}$, $PAI_{VR50\text{-}pheV\text{-}2}$) were integrated together with a second PAI at the respective integration site. (c) Comparison of selected *iuc*-containing PAIs. The integrase gene, *iuc* locus, and selected virulence-associated genes on the islands are labeled. The gradient scale shows the level of nucleotide identity. Sequence comparisons were performed using EasyFig[10].

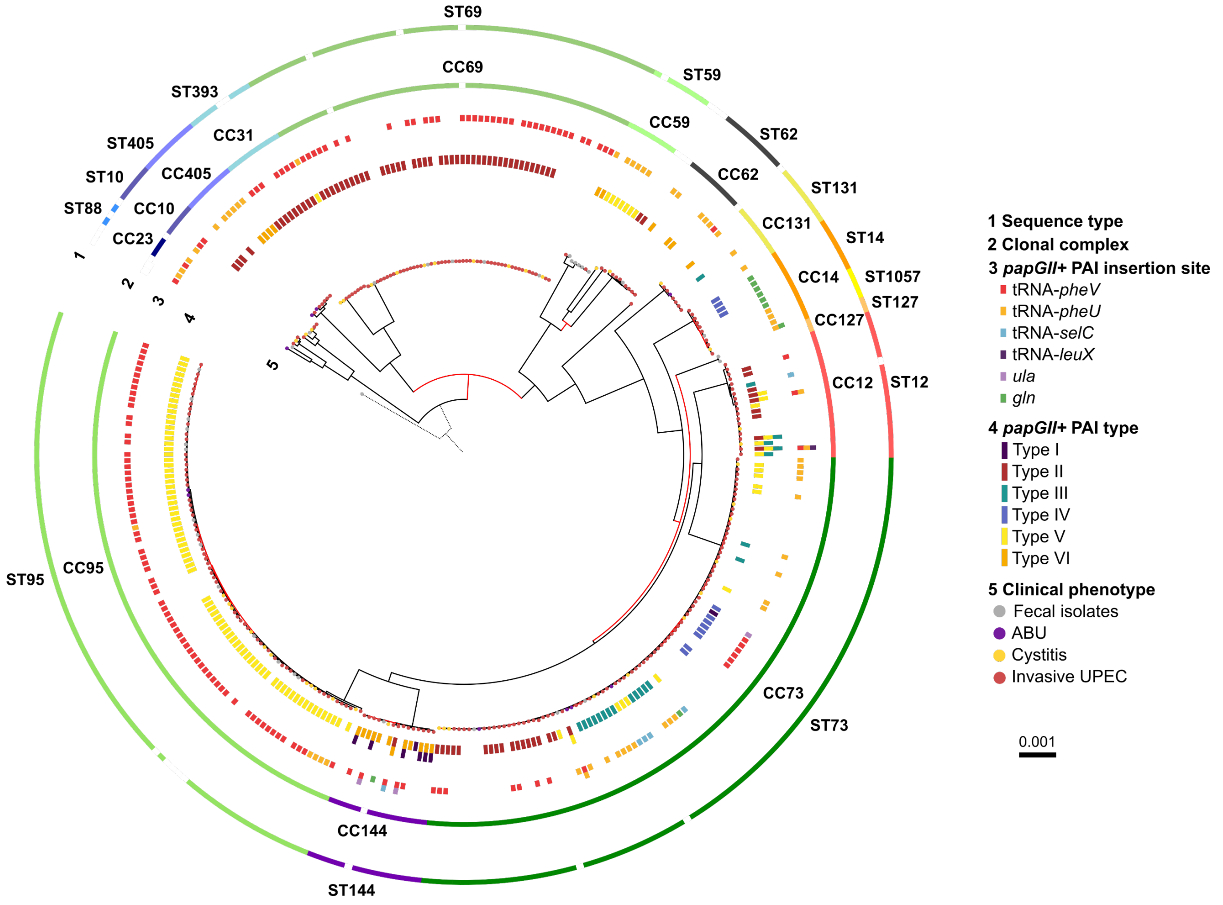

Supplementary Fig. 16. Phylogenetic tree of *papGII*+ isolates.

Maximum-likelihood phylogenetic tree of 333 *papGII*+ isolates and isolate 495_PUTI_Fec (clade I) as outgroup based on 192,889 variable sites identified in a core genome alignment (2.573 Mbp). Clinical phenotypes of isolates are indicated at branch tips. Dominant clonal complexes (CC) and corresponding sequence types (STs), *papGII*-containing pathogenicity island (*papGII*+ PAI) insertions sites, and *papGII*+ PAI types are annotated. Some isolates carried more than one *papGII*+ PAI. *papGII*+ PAI type and insertion site could not be identified for all isolates (no symbol). The *papGII*+ PAI types were either identified in high-quality assemblies or predicted by mapping reads to resolved islands. The *papGII*+ PAI integration sites could not be detected in highly fragmented assemblies. The scale bar indicates the number of substitutions per site. Nodes with bootstrap values <70 are labeled in red. The branch length of the outgroup was reduced (dashed line). The tree was visualized using iTOL[12]. ABU: asymptomatic bacteriuria

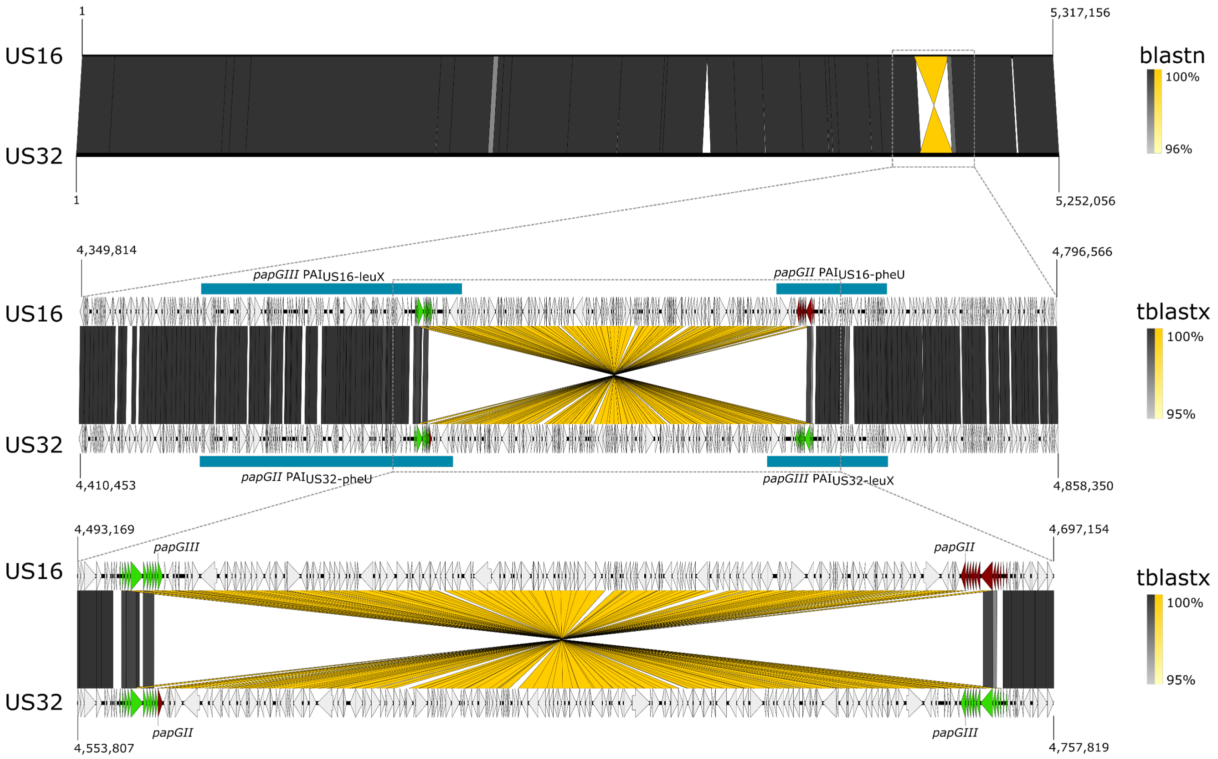

Supplementary Fig. 17. Synteny plots of whole genomes and genomic subregions of isolates US16 and US32 depicting a large chromosomal inversion between two *pap* sites.

US16 and US32 are part of *papGII*+ lineage CC73-L1. Isolates of this sublineage carry complete *papGII* and *papGIII* operons in inversed orientation on distinct pathogenicity islands (PAIs) integrated at the tRNA-*pheU* and tRNA-*leuX* integration sites, respectively. The depicted inversion of a 167 kb fragment between the two *pap* operons included the tRNA-*pheU* and tRNA-*leuX* genes, respective PAI integrase genes and *papG* alleles, and suggested a recombination event with the *pap* loci acting as inverted repeats. The *papGIII*$_{\text{US16}}$-associated *papIBAHCDJKE* genes were duplicated into both *pap* loci of US32. The *papGII* gene in US32 was hence associated with a different *papA* allele than in US16 and was integrated into a different PAI. The gene *papA* encodes the immunogenic major fimbrial subunit PapA. Isolate 194-Pyelo of the same sublineage carried a PAI (PAI$_{\text{194-pheU}}$) that shared all genes with PAI$_{\text{US32-pheU}}$ but contained a *papF* allele identical to the *papGII* locus in US16, suggesting an independent recombination event. PAIs are labeled (blue bars) and *papGII*-associated genes (red) and *papGIII*-associated genes (green) colored according to their association in US16. Chromosomal positions of the subregions are labeled. The gradient scale shows the level of nucleotide (BLASTn) or amino acid (tBLASTx) identity. Sequence comparisons were performed using EasyFig[10].

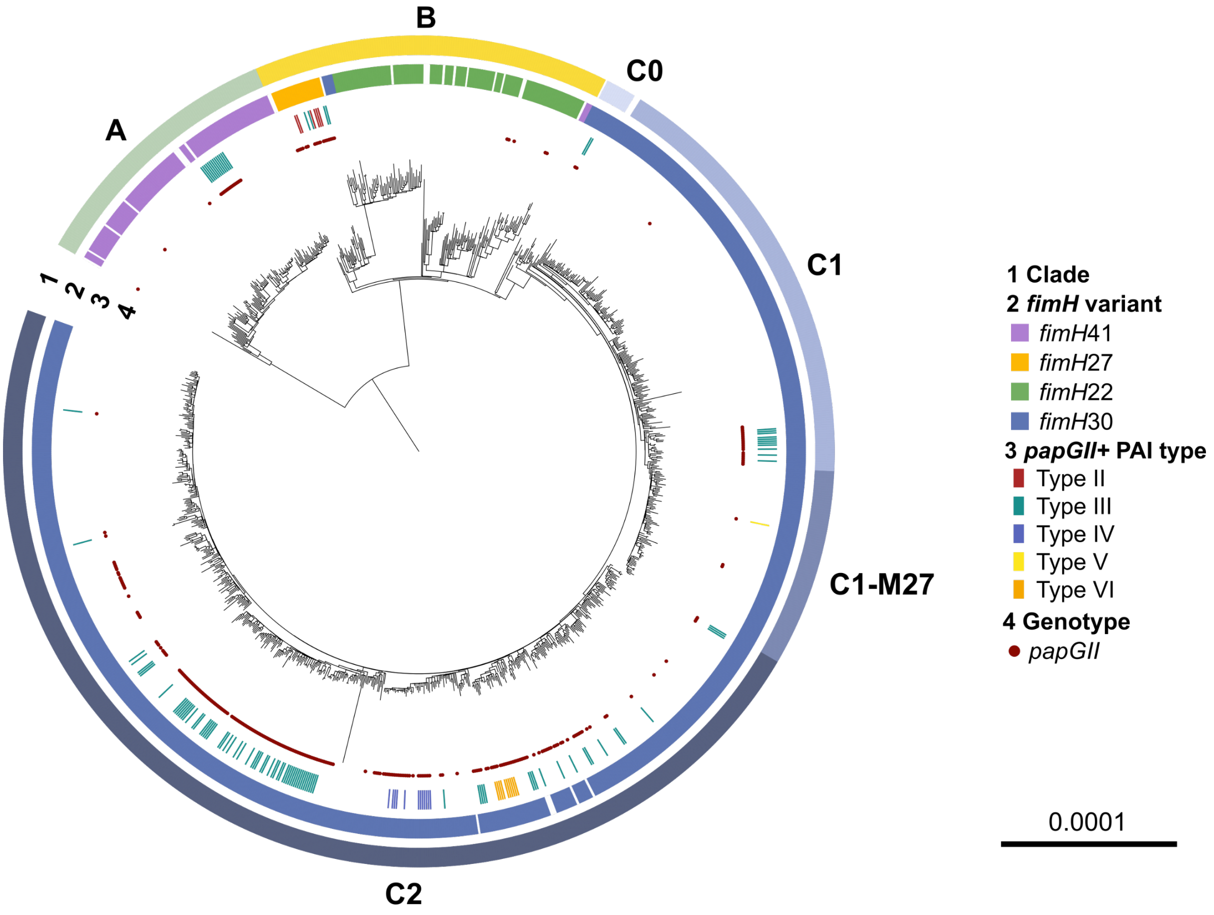

Supplementary Fig. 18. ***papGII*+ isolates and *papGII*+ pathogenicity island (PAI) types within pandemic uropathogenic *E. coli* (UPEC) lineage CC131 (ST131).**

Mid-point rooted maximum-likelihood phylogeny inferred from 25,716 variable sites in a 2.563 Mbp core genome alignment of 1,076 *E. coli* isolates belonging to CC131. The dataset consisted of 59 assemblies from the main dataset pooled with 1,017 additional publicly available assemblies. CC131 encompasses the three main clades A, B, and C, which are differentiated by dominant *fimH* alleles (clade A: *fimH*41; clade B: *fimH*22 and *fimH*27; clade C: *fimH*30). Clade C is subdivided into clades C0, clade C1 (*H*30R), and clade C2 (*H*30Rx)[18]. Phylogenetic clades (ring 1), presence of *fimH* variants (ring 2), *papGII*+ PAI types (ring 3), and presence of *papGII* (ring 4) are shown. For 137 out of 281 CC131 *papGII*+ isolates, the *papGII*+ PAI type could be identified in high-quality assemblies or predicted using read-mapping. The scale bar indicates the number of substitutions per site. The tree was visualized using iTOL[12].

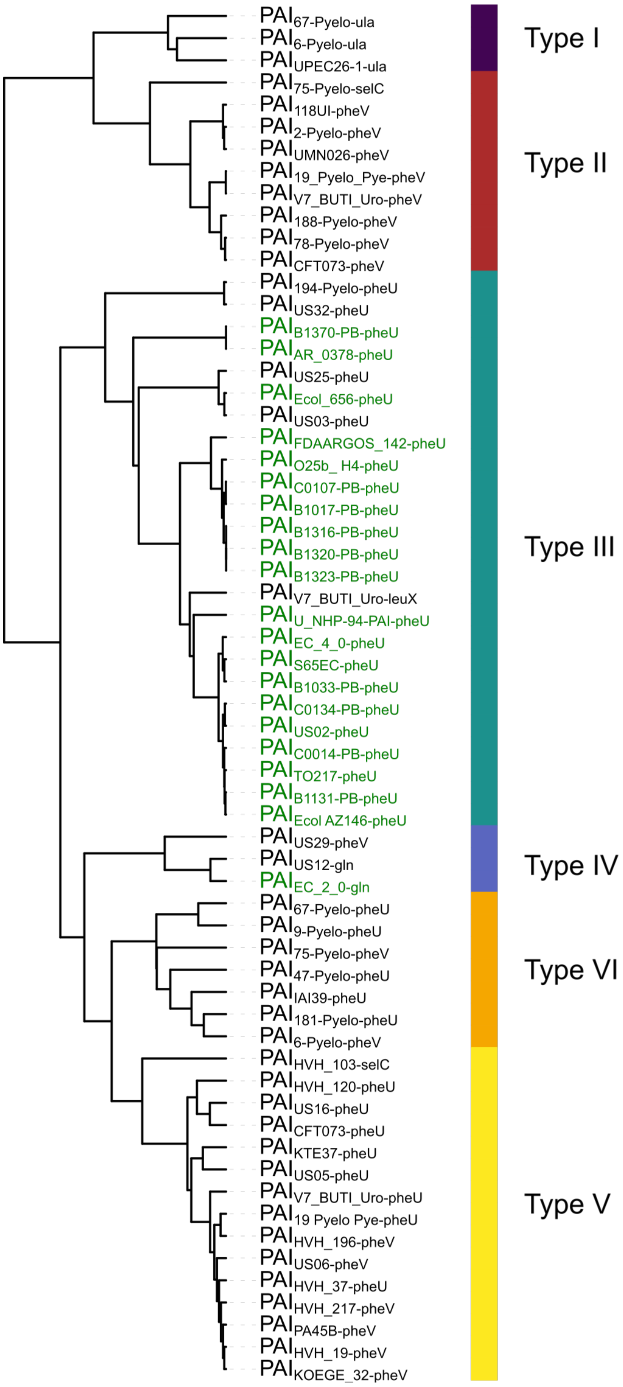

Supplementary Fig. 19. Clustering of resolved *papGII*-containing pathogenicity islands (PAIs) including PAIs identified in an extended dataset of CC131 isolates.

The 62 resolved *papGII*+ PAIs identified in high-quality genome assemblies were hierarchically clustered based on pairwise mash distance. These included the 42 PAIs identified in genomes of the main dataset (Supplementary Fig. 8b) and 20 PAIs identified in genomes of the CC131 dataset (highlighted in green). *papGII*+ PAI types are annotated.

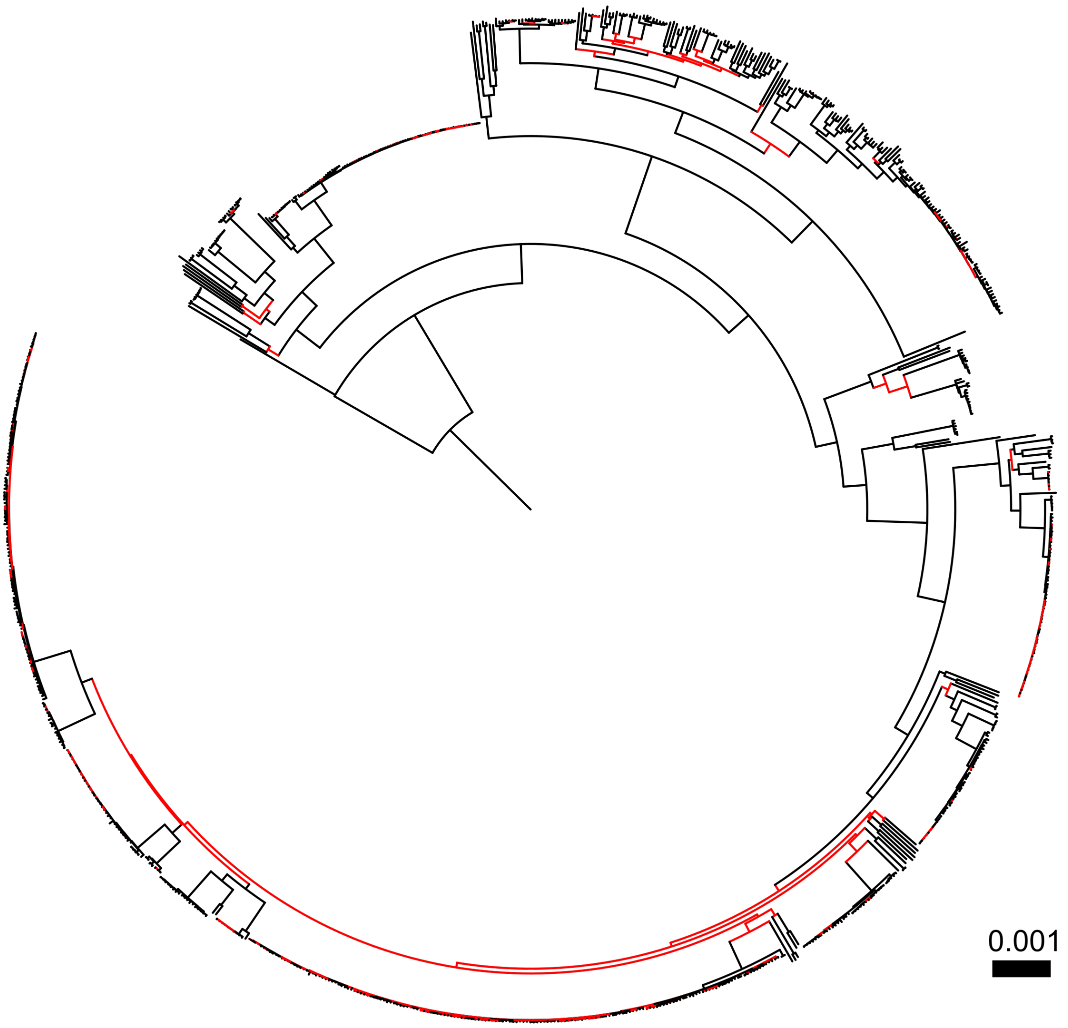

Supplementary Fig. 20. Bootstrap values of the phylogenetic tree of 907 *E. coli* isolates.

Midpoint-rooted maximum-likelihood phylogenetic tree with 100 bootstrap replicates based on 109,023 variable sites identified in a core genome alignment (1.136 Mbp). Red-colored branches indicate nodes with bootstrap values of <70. The scale bar indicates the number of substitutions per site. The tree was visualized using iTOL[12].

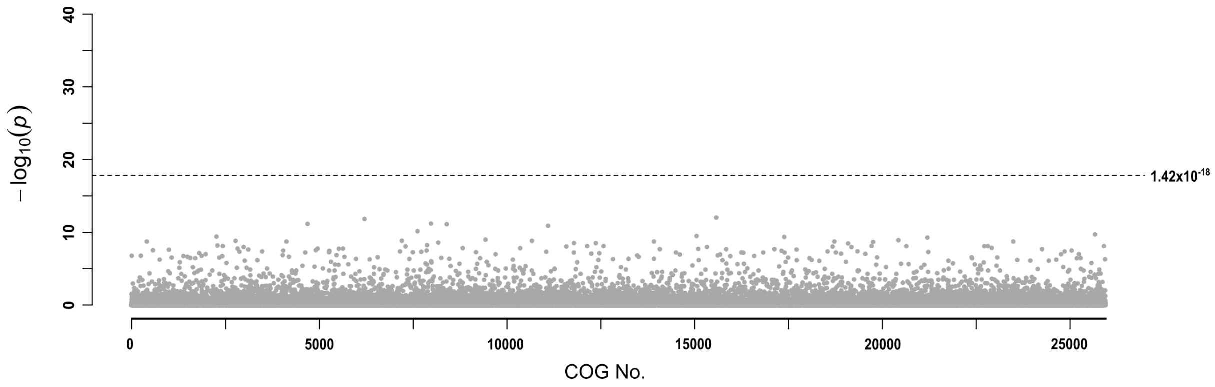

Supplementary Fig. 21. Manhattan plot for pan-genome wide associations for invasive vs. non-invasive uropathogenic *E. coli* (UPEC): clusters of orthologues genes (COGs) absent in reference strain UMN026.

Data are based on 30,705 COGs identified in 722 UPEC isolates. Each dot represents one COG. Fecal isolates were not considered in the analysis, as their clinical urinary phenotype is unknown. The vertical axis gives raw *P* values of Fisher's exact statistics (two-sided). To account for the effects of sample size and population structure, the genome-wide significance threshold (dotted line, raw $P = 1.42 \times 10^{-18}$) was inferred from a simulated dataset using treeWAS. The plot shows *P* values of 25,941 COGs of the UPEC pan-genome that were not identified in reference strain UMN026 (GCA_000026325.2). A Manhattan plot of COGs identified in UMN026 is shown in Fig. 2. The horizontal axis gives a number consecutively assigned to each COG.

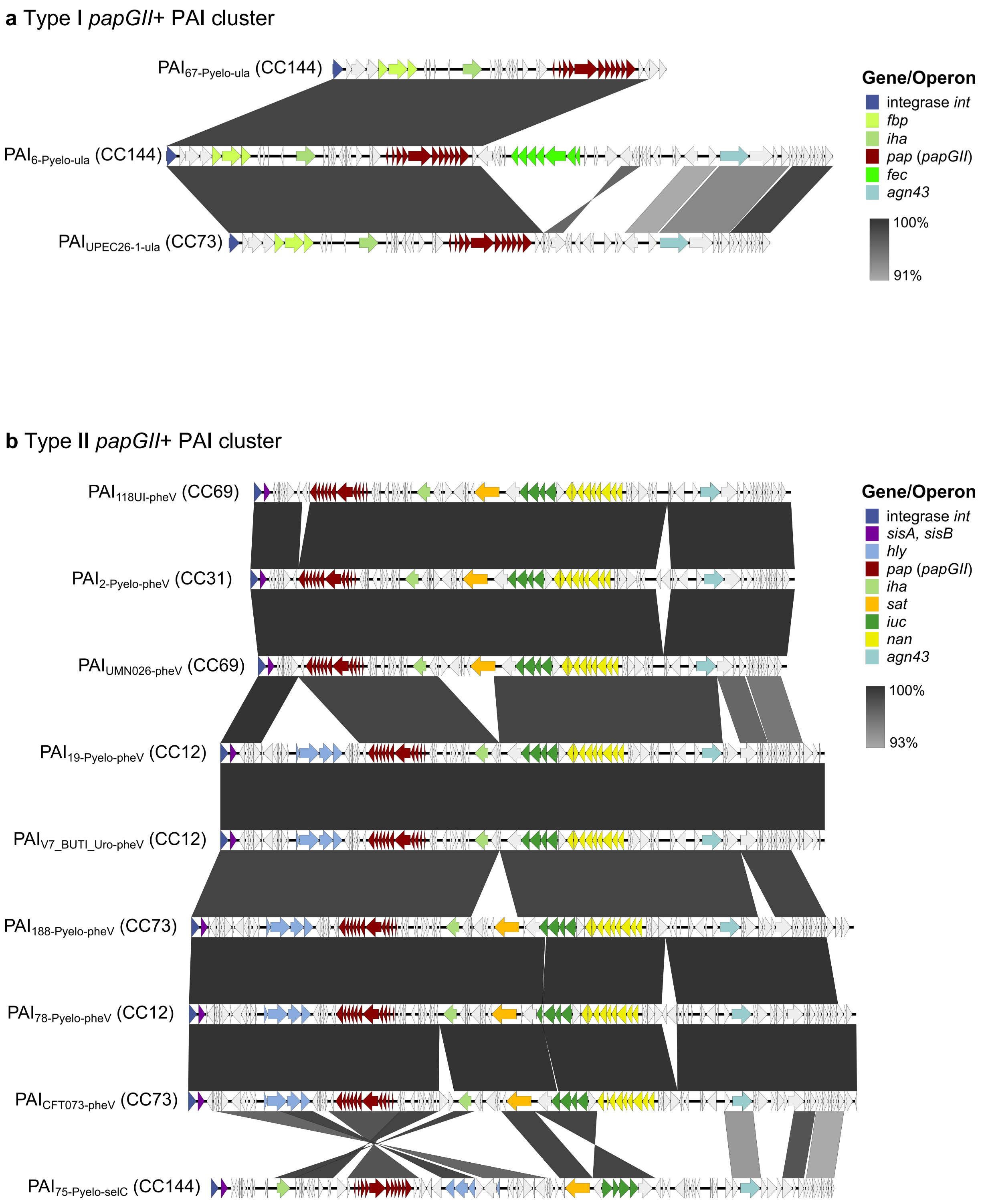
a Type I *papGII*+ PAI cluster
$PAI_{67\text{-Pyelo-ula}}$ (CC144)
$PAI_{6\text{-Pyelo-ula}}$ (CC144)
$PAI_{UPEC26\text{-}1\text{-ula}}$ (CC73)
Gene/Operon
integrase *int*
fbp
iha
pap (*papGII*)
fec
agn43
100%
91%
b Type II *papGII*+ PAI cluster
$PAI_{118UI\text{-pheV}}$ (CC69)
$PAI_{2\text{-Pyelo-pheV}}$ (CC31)
$PAI_{UMN026\text{-pheV}}$ (CC69)
$PAI_{19\text{-Pyelo-pheV}}$ (CC12)
$PAI_{V7\_BUTI\_Uro\text{-pheV}}$ (CC12)
$PAI_{188\text{-Pyelo-pheV}}$ (CC73)
$PAI_{78\text{-Pyelo-pheV}}$ (CC12)
$PAI_{CFT073\text{-pheV}}$ (CC73)
$PAI_{75\text{-Pyelo-selC}}$ (CC144)
Gene/Operon
integrase *int*
sisA, sisB
hly
pap (*papGII*)
iha
sat
iuc
nan
agn43
100%
93%

c Type III *papGII*+ PAI cluster

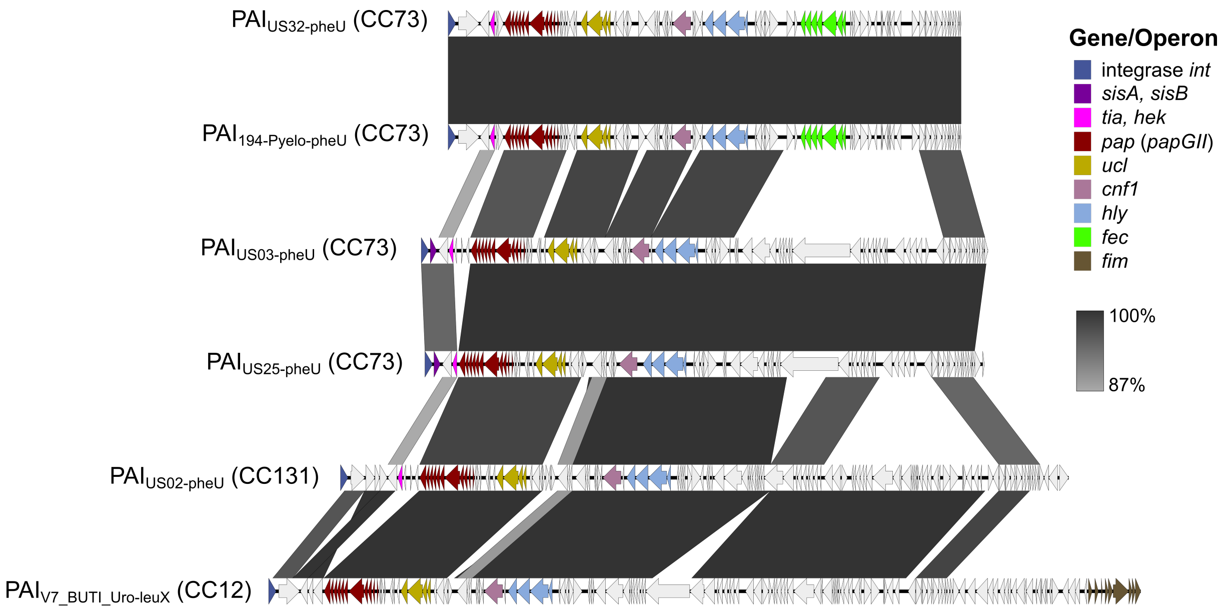

d Type IV *papGII*+ PAI cluster

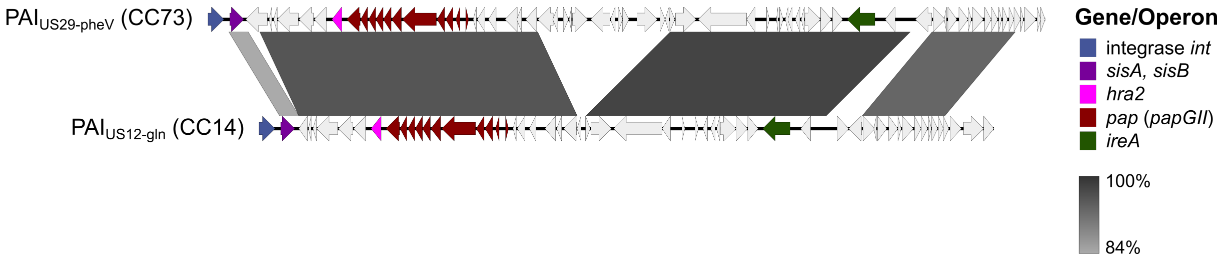

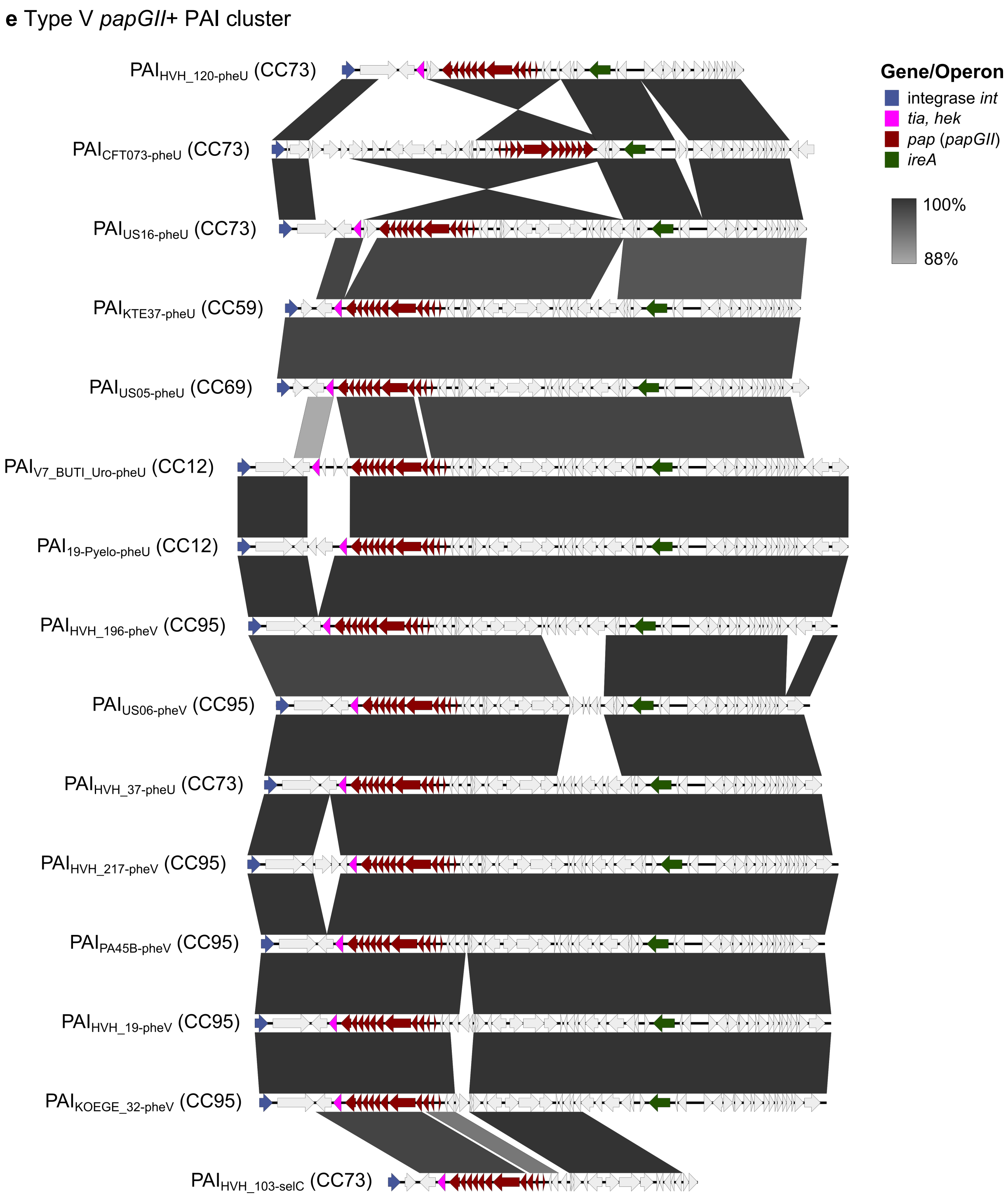
e Type V papGII+ PAI cluster
PAIHVH_120-pheU (CC73)
PAICFT073-pheU (CC73)
PAIUS16-pheU (CC73)
PAIKTE37-pheU (CC59)
PAIUS05-pheU (CC69)
PAIV7_BUTI_Uro-pheU (CC12)
PAI19-Pyelo-pheU (CC12)
PAIHVH_196-pheV (CC95)
PAIUS06-pheV (CC95)
PAIHVH_37-pheU (CC73)
PAIHVH_217-pheV (CC95)
PAIPA45B-pheV (CC95)
PAIHVH_19-pheV (CC95)
PAIKOEGE_32-pheV (CC95)
PAIHVH_103-selC (CC73)
Gene/Operon
integrase int
tia, hek
pap (papGII)
ireA
100%
88%

f Type VI *papGII*+ PAI cluster

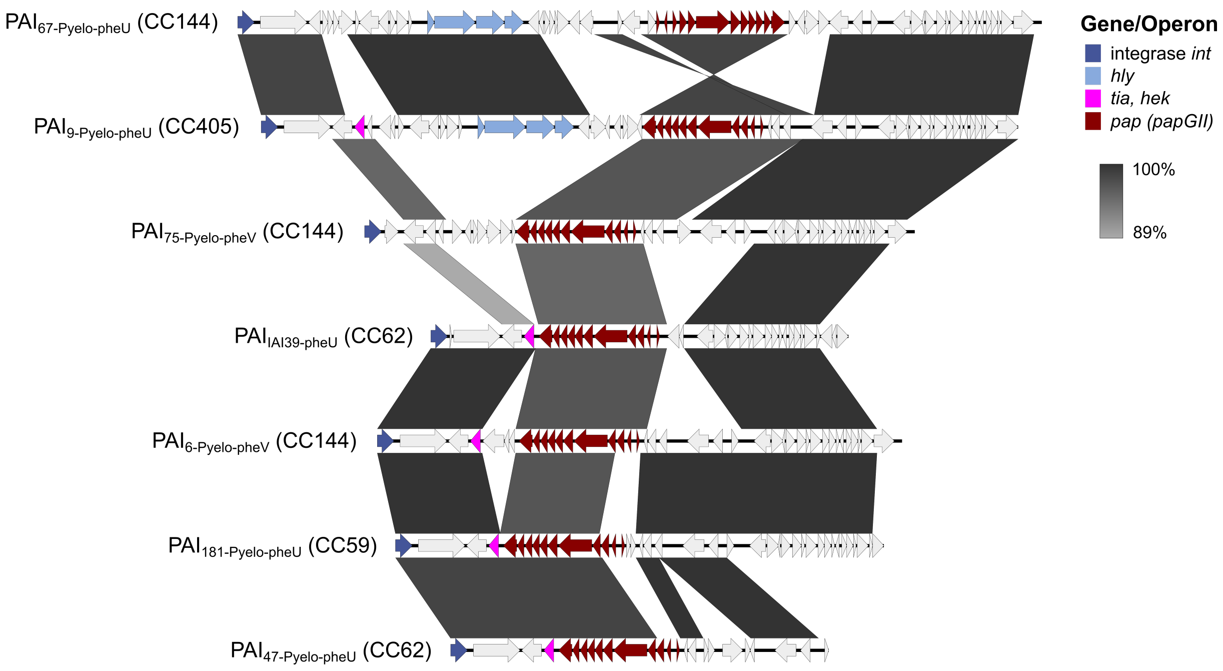

Supplementary Fig. 22. Genetic organization of 42 resolved *papGII*-containing pathogenicity islands (*papGII*+ PAIs).

PAIs are grouped into the six clusters (a) type I, (b) type II, (c) type III (d), type IV, (e) type V, and (f) type VI *papGII*+ PAI. The *pap* operon, integrase gene, and identified virulence-associated genes are labelled. The gradient scale shows the level of nucleotide identity. PAI sequences were compared and visualized using EasyFig[10].

Supplementary Tables

Supplementary Table 1. Description of the 16 source collections included in the main dataset of this study.

The table is continued on the following page. ABU: asymptomatic bacteriuria, UTI: urinary tract infection, UPEC: uropathogenic *E. coli*, na: not available

Collection	Clinical phenotype	Clinical phenotype grouped	Population	Host age (median)	Geographic origin	Year of collection	Reference
LtABU	long-term ABU	non-invasive UPEC	older adults of long-term care facilities	70 - 97 (86)	Ghent and Antwerp, Belgium	2017 - 2018	this study
dsABU	ABU	non-invasive UPEC	female individuals with diabetes	12 - 25 (19)	Pécs, Hungary	2010 - 2012	Stork et al.[19]
RT_ABU	ABU	non-invasive UPEC	renal transplant recipients	29 - 74 (54)	Brussels, Belgium	2012 - 2015	Coussement et al.[20]
MVAST_ABU	ABU	non-invasive UPEC	community-based male veterans	38 - 92 (69)	Minneapolis, USA	2010 - 2011	this study; Drekonja et al.[21]
Koege_cys	cystitis	non-invasive UPEC	male and female patients	3 - 81 (61)	Copenhagen, Denmark	2005 - 2006	Skjøt-Rasmussen et al.[22]
KTE_cys	cystitis	non-invasive UPEC	female patients	19 – 53 (34)	Zealand, Denmark	2009 - 2010	Nielsen et al.[23]
PUTI_cys	cystitis	non-invasive UPEC	female patients	na	Minneapolis, USA	1999 - 2000	Johnson et al.[24] Sannes et al.[25]
UMEA_cys	cystitis	non-invasive UPEC	female patients	17 - 85 (48)	Sweden (Multicenter)	1995 - 1997	Ejrnæs et al.[26]
Rec_cys	recurrent cystitis	non-invasive UPEC	female patients	18 - 49 (na)	Seattle, USA	2003 - 2006	Czaja et al.[27]; Schreiber et al.[17]
MC_pye	acute uncomplicated pyelonephritis	invasive UPEC	non-compromised premenopausal female patients, community-acquired	>18	USA (Multicenter)	1994 - 1997	this study; Johnson et al.[24]; Sannes et al.[25,28], Talan et al.[29]
HVH_urb	urinary-source bacteremia	invasive UPEC	community- and hospital acquired	19 - 102 (79)	Copenhagen, Denmark	2003 - 2005	Skjøt-Rasmussen et al.[30]
UHS_urb	urinary-source bacteremia	invasive UPEC	non-compromised adult patients	19 - 96 (70)	Southampton, UK	2015 - 2016	Dale et al.[31]
BUTI_uro	urinary-source bacteremia	invasive UPEC	non-compromised (n = 21) and compromised (n = 46) patients	20 - 91 (62)	Seattle, USA	1981 - 1985	Johnson et al.[15,24]
UZA_uro	urosepsis	invasive UPEC	non-compromised patients, community-acquired	0 - 92 (75)	Antwerp, Belgium	2015 - 2017	this study
KTE_fec	fecal	fecal	healthy female individuals who never had a UTI and non-UTI-associated clones from female patients	18 – 53 (37)	Zealand, Denmark	2009 - 2010	Nielsen et al.[23]
MN_fec	fecal	fecal	female individuals without UTI symptoms or acute infections	na	Minneapolis, USA	1996 - 2000	Johnson et al.[24]; Sannes et al.[25,32]

Supplementary Table 1 continued

Collection	No. isolates	Data source	Data type	Sequencing technology	No. isolates in the original collection	Exclusion of isolates for this study
LtABU	43	in-house sequenced	read data	Illumina MiSeq	43	
dsABU	9	NCBI SRA	read data	Illumina MiSeq	9	
RT_ABU	19	NCBI SRA	read data	Illumina MiSeq	19	
MVAST_ABU	39	in-house sequenced	read data	Illumina MiSeq	39	
Koege_cys	19	NCBI SRA	read data	Illumina HiSeq 2000 (including paired-end jumping libraries)	20	ambiguous taxonomic classification (n = 1)
KTE_cys	48	NCBI SRA	read data	Illumina HiSeq 2000 (including paired-end jumping libraries)	48	
PUTI_cys	30	NCBI SRA	read data	Illumina Genome Analyzer IIx	39	low sequence quality (n = 1), inconclusive metadata (n = 1), sampling bias correction (n = 7)
UMEA_cys	105	NCBI SRA	read data	Illumina HiSeq 2000 (including paired-end jumping libraries)	107	low sequence quality (n = 1), corrupted read files (n = 1)
Rec_cys	15	NCBI	assemblies	Illumina HiSeq 2000	43	same-clone isolates (24), low sequence quality (n = 4)
MC_pye	70	in-house sequenced (n = 39) and NCBI SRA (n = 31)	read data	Illumina MiSeq and Genome Analyzer IIx	79	low sequence quality (n = 4), sampling bias correction (n = 5)
HVH_urb	190	NGDC GSA	read data	Illumina HiSeq 2000 (including paired-end jumping libraries)	196	low sequence quality (n = 5), corrupted read files (n = 1)
UHS_urb	22	NCBI SRA	read data	Illumina HiSeq 2000	23	low sequence quality (n = 1)
BUTI_uro	67	NCBI SRA	read data	Illumina Genome Analyzer IIx	67	
UZA_uro	30	in-house sequenced	read data	Illumina MiSeq	30	
KTE_fec	102	NCBI SRA	read data	Illumina HiSeq 2000 (including paired-end jumping libraries)	148	read files not available (n = 6), same-clone isolates (n = 40)
MN_fec	81	NCBI SRA	read data	Illumina HiSeq 2000	116	low sequence quality (n = 9), inconclusive metadata (n = 3), sampling bias correction (n = 23)

Supplementary Table 2. Distribution of isolates associated with different clinical phenotypes or genotypes among phylogroups. ABU: asymptomatic bacteriuria, UPEC: uropathogenic *E. coli*

	A	B1	B2	C	D	E or cladeI	F	G
Clinical phenotype								
Fecal isolates (n = 185)	40 (21.6%)	28 (15.1%)	76 (41.1%)	0 (0%)	28 (15.1%)	2 (1.1%)	9 (4.9%)	2 (1.1%)
UPEC isolates (n = 722)	59 (8.2%)	35 (4.8%)	465 (64.4%)	25 (3.5%)	107 (14.8%)	8 (1.1%)	17 (2.4%)	6 (0.8%)
Non-invasive UPEC (n = 337)	46 (13.6%)	27 (8%)	201 (59.6%)	8 (2.4%)	38 (11.3%)	7 (2.1%)	6 (1.8%)	4 (1.2%)
ABU (n = 113)	14 (12.4%)	12 (10.6%)	65 (57.5%)	4 (3.5%)	11 (9.7%)	1 (0.9%)	2 (1.8%)	4 (3.5%)
Cystitis (n = 224)	32 (14.3%)	15 (6.7%)	136 (60.7%)	4 (1.8%)	27 (12.1%)	6 (2.7%)	4 (1.8%)	0 (0.0%)
Invasive UPEC (n = 385)	13 (3.4%)	8 (2.1%)	264 (68.6%)	17 (4.4%)	69 (17.9%)	1 (0.3%)	11 (2.9%)	2 (0.5%)
Genotype								
papGII+ isolates (n = 333)	6 (1.8%)	2 (0.6%)	230 (69.1%)	3 (0.9%)	71 (21.3%)	0 (0%)	20 (6.0%)	1 (0.3%)
Other (n = 574)	93 (16.2%)	61 (10.6%)	311 (54.2%)	22 (3.8%)	64 (11.1%)	10 (1.7%)	6 (1.0%)	7 (1.2%)

Supplementary Table 3. Genome-wide significant determinants for invasive vs. non-invasive uropathogenic *E. coli* (UPEC) identified using DBGWAS.

DBGWAS for 385 invasive UPEC isolates and 337 non-invasive UPEC isolates identified 27 components (compacted De Brujin graphs) containing nodes with q values <0.05. Each row in the table shows characteristics of the node with the lowest q value within each component, including frequencies, nucleotide sequence, the gene or region the sequence was identified in, and a possible presence of the node on *papGII*-containing pathogenicity islands (PAI). q values represent Benjamini-Hochberg transformed *P* values controlling for false discovery rate. Genomes of strains UMN026 (GCA_000026325.2), CFT073 (GCA_000007445.1), US07 (GCA_014140745.1), US29 (GCA_014131555.1), ATCC700415 (GCA_003491265.1), and UMEA_3155 (GCA_000460395.1) were used as annotation reference.

Rank	Sequence of node with the lowest q value within component	Frequency invasive UPEC isolates (n = 385)	Frequency non-invasive UPEC isolates (n = 337)	q	Gene	Product/Region	Reference strain for annotation	Identified on *papGII+* PAIs
1	GGAGAGACAACGATGAGATGGTACAAAGCAGGTACACAAA	244 (63.4%)	50 (14.8%)	8.56E-13	*papGII*	P fimbriae adhesin variant PapGII	UMN026	yes
2	CCCGCTATTACCTGTTATTACTGGAAAAAGCA	223 (57.9%)	44 (13.1%)	3.07E-09	*papI*	*pap* operon regulatory protein	UMN026	yes
3	AAAAAAGTAAGATGACTGTGCGAAGGTGGCGGAATTG	298 (77.4%)	318 (94.4%)	2.62E-04	tRNA-*leu*(cag)	tRNA-Leu	UMN026	no
4	TCATCAGTGTGACGACTGCGGCGGCCATCCATCCA	242 (62.9%)	107 (31.8%)	2.52E-03	*insD*	IS3-like element IS2 family transposase	UMN026	yes
5	AGTACGGGGTCGGACCGGTATGCAGTGAACT	185 (48.1%)	42 (12.5%)	3.13E-03	*ins*	Putative transposase OrfB	UMN026	yes
6	ATTCAGAAGCTGGTACAGAGTGGCGGAAATCAGAAAGAC	107 (27.8%)	21 (6.2%)	4.34E-03	*papE*	P fimbriae minor subunit	UMN026	yes
7	CGATAACCAGCGCCTGTTCCGCCATCTTGAGCCTGCGGG	194 (50.4%)	70 (20.8%)	9.59E-03	*iucB*	Aerobactin biosynthesis protein	UMN026	yes
8	AGAACAGCGGCTTCACCCGGCTTTTTGTCTGAA	191 (49.6%)	71 (21.1%)	0.01	*yfjJ*	Inovirus Gp2 family protein	CFT073	yes
9	TGCCTGATTTCTGTATAACTGAGATATTCCTACACAGCAGACA	108 (28.1%)	20 (5.9%)	0.01	-	Intergenic region between *papGII* and *papX*	UMN026	yes
10	CACGGTAAATAAATCCGACCGGCAGATCCCCAAGCATCTG	282 (73.2%)	125 (37.1%)	0.02	*iucA*	Aerobactin biosynthesis protein	UMN026	yes
11	GAATATACGCGAGAGCGTACTGTTGCCCGGGTCTATGTCTGAA ATGCATTTTTTTT	122 (31.7%)	21 (6.2%)	0.02	*papB*	*pap* operon regulatory protein	UMN026	yes
12	CACTAACATAGTTAGTTGTAGTATCCAGCGTAGTAT	206 (53.5%)	223 (66.2%)	0.02	-	Intergenic region tRNA-Lys(ttt) and tRNA-Val(tac)	CFT073	no
13	TCACAGGTTCGAATCCCGTCGTAGCCACCAA	373 (96.9%)	297 (88.1%)	0.02	tRNA-*met*(cat)	tRNA-Met	UMN026	no
14	ACCTGCCCGGAACACCACCGTGACACCGGAGAT	99 (25.7%)	116 (34.4%)	0.02	hypothetical	Host specificity protein J	CFT073	no
15	ACCACTTCCGTTTCATAGCCCAGCGCCTCAACCTGCTCT	28 (7.3%)	4 (1.2%)	0.02	*traI*	Conjugation protein TraI	US07	no; part of *iuc*-containing IncF plasmid
16	AGCTACCAGGCTGCTCCACCCCGCGTCACCG	296 (76.9%)	307 (91.1%)	0.02	tRNA-*met*(cat)	tRNA-Met	UMN026	no
17	TACGACACATAGATTTTGACGGGTACGTCAGCGGCGGTAGCCA GCATC	22 (5.7%)	3 (0.9%)	0.02	hypothetical	AAA family ATPase	US29	no; part of IncF plasmid
18	ACAACGTTTATCGACCTCACACGAACAACGAT	184 (47.8%)	178 (52.8%)	0.02	*ins*	IS element	ATCC700415	no
19	CCGGTTCTGGTGCAGAAGCTGCAGATGACGAA	161 (41.8%)	52 (15.4%)	0.03	hypothetical	DUF957-domain containing protein	CFT073	yes
20	AGTTGTGGTGCGGCGATAAATATCTGATCCAGCGC	193 (50.1%)	72 (21.4%)	0.03	*iucB*	Aerobactin biosynthesis protein	UMN026	yes
21	AAATGGATAGACATAGGGGGATTTTTGGCTTTATT	12 (3.1%)	35 (10.4%)	0.03	hypothetical	DUF2931-containing protein	UMEA_3155	no
22	AACCATACCTTGAGTCATACATCGACCCCGGACAAGCACATGC ACCTGTATCACTA	172 (44.7%)	77 (22.8%)	0.03	*pilV*	Conjugative transfer pilus tip adhesin PilV	CFT073	no
23	CAGACAGTAACGCACCGGATCCCTGCAAAATGGTACC	83 (21.6%)	20 (5.9%)	0.03	-	Intergenic region	UMN026	yes
24	GAGTGCTCCGGTACCCATGCGCCCTCCTGACGTGGCACCTGAG	30 (7.8%)	8 (2.4%)	0.03	*traI*	Conjugation protein TraI	US07	no; part of *iuc*-containing IncF plasmid
25	ATCGAGATTGCGGGTGATGGCTTTCATTGCTG	135 (35.1%)	32 (9.5%)	0.03	hypothetical	DUF4942-domain containing protein	CFT073	yes
26	CCGGACACTTTATCGTCAGCTGCTACCGGAAC	142 (36.9%)	41 (12.2%)	0.03	*yfjP*	GTPase	CFT073	yes
27	CATCAGTAAACTGGATAACTTTCTCGGTCAGCTTGATGGTATCC TTCGCC	255 (66.2%)	104 (30.9%)	0.04	hypothetical	Putative phosphoethanolamine transferase	UMN026	yes

Supplementary Table 4. Frequency of *papGII* among invasive and non-invasive UPEC isolates stratified by host age (a) and gender (b).

Frequencies, *P* values (two-sided Fisher's exact test), and odds ratios with 95% confidence intervals (CI) are shown for 573 out of 722 UPEC isolates with available metadata on host age (a) and 592 out of 722 UPEC isolates with available metadata on host gender (b).

(a)

Host age	*papGII* frequency among invasive UPEC isolates	*papGII* frequency among non-invasive UPEC isolates	*P*	Exact *P*	Odds ratio (95% CI)
<30	11/13 (84.6%)	10/53 (18.9%)	<0.001	1.77E-05	22.1 (4.0 - 235.4)
30 - 60	42/60 (70.0%)	23/112 (20.5%)	<0.001	2.88E-10	8.9 (4.2 - 19.8)
>60	128/214 (59.8%)	11/121 (9.1%)	<0.001	1.52E-21	14.8 (7.4 - 32.3)

(b)

Host gender	*papGII* frequency among invasive UPEC isolates	*papGII* frequency among non-invasive UPEC isolates	*P*	Exact *P*	Odds ratio (95% CI)
female	123/185 (66.5%)	39/250 (15.6%)	<0.001	4.60E-28	10.7 (6.6 - 17.5)
male	58/103 (56.3%)	7/54 (13.0%)	<0.001	7.58E-08	8.5 (3.4 - 24.6)

Supplementary Table 5. Characteristics of resolved *papGII*-containing pathogenicity islands (*papGII*+ PAIs).

The 42 PAIs were identified in high-quality assemblies of 35 isolates of the main dataset. The number of phage genes and putative complete or incomplete phage regions were predicted using PHASTER. VAG: virulence-associated gene, VF: virulence factor

PAI	Isolate	Insertion site	*papGII*+ PAI type	Size (kb)	No. genes	No. VAGs	No. VFs	No. phage genes	No. phage regions
PAI$_{\text{19-Pyelo-pheU}}$	19-Pyelo	*pheU*	Type V	58	59	13	3	24	2
PAI$_{\text{CFT073-pheU}}$	CFT073	*pheU*	Type V	51	59	12	2	25	2
PAI$_{\text{HVH 103-selC}}$	HVH_103	*selC*	Type V	29	31	13	3	0	0
PAI$_{\text{HVH 120-pheU}}$	HVH_120	*pheU*	Type V	38	41	13	3	13	1
PAI$_{\text{HVH 19-pheV}}$	HVH_19	*pheV*	Type V	55	58	13	3	23	2
PAI$_{\text{HVH 196-pheV}}$	HVH_196	*pheV*	Type V	56	57	13	3	24	2
PAI$_{\text{HVH 217-pheV}}$	HVH_217	*pheV*	Type V	56	58	13	3	23	2
PAI$_{\text{HVH 37-pheU}}$	HVH_37	*pheU*	Type V	53	56	13	3	24	2
PAI$_{\text{KOEGE 32-pheV}}$	KOEGE_32	*pheV*	Type V	54	57	13	3	23	2
PAI$_{\text{KTE37-pheU}}$	KTE37	*pheU*	Type V	49	55	14	4	24	2
PAI$_{\text{PA45B-pheV}}$	PA45B	*pheV*	Type V	53	57	13	3	24	2
PAI$_{\text{US05-pheU}}$	US05	*pheU*	Type V	50	54	14	4	22	2
PAI$_{\text{US06-pheV}}$	US06	*pheV*	Type V	51	55	13	3	24	2
PAI$_{\text{US16-pheU}}$	US16	*pheU*	Type V	50	54	13	3	20	2
PAI$_{\text{V7 BUTI Uro-pheU}}$	V7_BUTI_Uro	*pheU*	Type V	58	60	14	3	24	2
PAI$_{\text{118UI-pheV}}$	118UI	*pheV*	Type II	84	86	22	6	36	4
PAI$_{\text{188-Pyelo-pheV}}$	188-Pyelo	*pheV*	Type II	104	103	26	7	35	4
PAI$_{\text{19-Pyelo-pheV}}$	19-Pyelo	*pheV*	Type II	95	96	25	6	22	2
PAI$_{\text{2-Pyelo-pheV}}$	2-Pyelo	*pheV*	Type II	85	86	22	6	25	3
PAI$_{\text{75-Pyelo-selC}}$	75-Pyelo	*selC*	Type II	98	100	25	6	28	3
PAI$_{\text{78-Pyelo-pheV}}$	78-Pyelo	*pheV*	Type II	105	106	25	7	44	5
PAI$_{\text{CFT073-pheV}}$	CFT073	*pheV*	Type II	105	104	26	7	34	4
PAI$_{\text{UMN026-pheV}}$	UMN026	*pheV*	Type II	83	84	22	6	25	3
PAI$_{\text{V7 BUTI Uro-pheV}}$	V7_BUTI_Uro	*pheV*	Type II	95	96	25	6	22	2
PAI$_{\text{181-Pyelo-pheU}}$	181-Pyelo	*pheU*	Type VI	37	41	12	2	14	1
PAI$_{\text{47-Pyelo-pheU}}$	47-Pyelo	*pheU*	Type VI	28	26	12	2	8	1
PAI$_{\text{67-Pyelo-pheU}}$	67-Pyelo	*pheU*	Type VI	61	63	16	2	26	2
PAI$_{\text{6-Pyelo-pheV}}$	6-Pyelo	*pheV*	Type VI	40	41	12	2	14	1
PAI$_{\text{75-Pyelo-pheV}}$	75-Pyelo	*pheV*	Type VI	41	47	13	3	14	1
PAI$_{\text{9-Pyelo-pheU}}$	9-Pyelo	*pheU*	Type VI	57	58	17	3	22	2
PAI$_{\text{IAI39-pheU}}$	IAI39	*pheU*	Type VI	31	37	12	2	20	1
PAI$_{\text{6-Pyelo-ula}}$	6-Pyelo	*ula*	Type I	73	76	24	5	17	2
PAI$_{\text{UPEC26-1-ula}}$	UPEC26-1	*ula*	Type I	59	62	17	4	20	2
PAI$_{\text{67-Pyelo-ula}}$	67-Pyelo	*ula*	Type I	36	37	16	3	0	0
PAI$_{\text{US12-gln}}$	US12	*gln*	Type IV	55	58	14	4	33	3
PAI$_{\text{US29-pheV}}$	US29	*pheV*	Type IV	62	70	14	4	45	3
PAI$_{\text{194-Pyelo-pheU}}$	194-Pyelo	*pheU*	Type III	86	93	28	6	19	2
PAI$_{\text{US02-pheU}}$	US02	*pheU*	Type III	122	124	23	5	29	2
PAI$_{\text{US03-pheU}}$	US03	*pheU*	Type III	95	89	25	7	20	2
PAI$_{\text{US25-pheU}}$	US25	*pheU*	Type III	94	88	24	7	21	2
PAI$_{\text{US32-pheU}}$	US32	*pheU*	Type III	86	93	28	6	19	2
PAI$_{\text{V7 BUTI Uro-leuX}}$	V7_BUTI_Uro	*leuX*	Type III	146	140	31	6	42	3

Supplementary Table 6. Distribution of virulence factor classes by clinical phenotype.

Average number of virulence-associated genes (VAGs) per isolate by functional class and corresponding *P* values (compared to invasive UPEC; two-sided Mann-Whitney U test, Bonferroni corrected) in (a) all phylogroups and (b) phylogroup B2. The average number of VAGs overall and in class "Adhesion and invasion" was additionally calculated excluding the genes of the *pap* operon, irrespective of *papG* adhesin allele. ABU: asymptomatic bacteriuria, UPEC: uropathogenic *E. coli*

a

Clinical phenotype	VAGs total	VAGs total *excl. pap*	Iron uptake	Immune evasion and modulation	Secretion systems/ Auto-transporter	Toxins	Adhesion and invasion	Adhesion and invasion *excl. pap*	Bacterio-cins	Flagella	Other
Fecal isolates (n = 185)	264.5**	261.0**	54.2**	8.9**	52.3	5.9**	67.5**	64.0	4.8**	58.7	13.8**
ABU (n = 113)	272.1**	268.3**	61.3**	9.2**	48.2*	7.3**	66.4**	62.7	5.7**	61.1	14.5
Cystitis (n = 224)	287.3**	281.5*	62.7**	10.0**	53.4	11.9	72.2	66.4**	7.0	57.5	14.3
**Invasive UPEC (n = 385)**	**299.7**	**290.8**	**69.2**	**12.1**	**53.2**	**12.3**	**72.6**	**63.8**	**7.9**	**59.6**	**14.7**

*significantly ($P < 0.05$, two-sided Mann-Whitney U test, Bonferroni corrected) increased/decreased compared to invasive UPEC
**significantly ($P < 0.001$, two-sided Mann-Whitney U test, Bonferroni corrected) increased/decreased compared to invasive UPEC

Exact *P* values

Clinical phenotype	VAGs total	VAGs total *excl. pap*	Iron uptake	Immune evasion and modulation	Secretion systems/ Auto-transporter	Toxins	Adhesion and invasion	Adhesion and invasion *excl. pap*	Bacterio-cins	Flagella	Other
Fecal isolates (n = 185)	1.34E-23	1.12E-20	7.01E-26	1.67E-07	1.00E+00	7.70E-17	4.86E-07	1.00E+00	3.20E-13	1.00E+00	5.72E-09
ABU (n = 113)	5.49E-17	6.55E-14	1.14E-06	1.26E-05	1.82E-02	1.93E-07	2.50E-06	1.00E+00	5.13E-05	1.00E+00	5.59E-01
Cystitis (n = 224)	8.22E-05	2.77E-03	1.00E-09	5.85E-06	1.00E+00	1.00E+00	1.00E+00	4.93E-04	1.53E-01	2.91E-01	5.47E-02
Invasive UPEC (n = 385)	-	-	-	-	-	-	-	-	-	-	-

b

Phylogroup B2

Clinical phenotype	VAGs total	VAGs total excl. *pap*	Iron uptake	Immune evasion and modulation	Secretion system/ Auto-transporter	Toxins	Adhesion and invasion	Adhesion and invasion *excl. pap*	Bacterio-cins	Flagella	Other
Fecal isolates (n = 76)	285.5**	279.8*	67.4**	13.8	49.7	12.1**	64.3**	58.6*	7.6*	57.3	15.5
ABU (n = 65)	284.7**	279.4**	69.8*	11.8*	42.4**	11.6**	63.2**	57.9**	6.6**	66.1*	15.2
Cystitis (n = 136)	299.6	291.9	69.7**	12.7*	49.3	18.5	72.4	64.7	9.2	54.3	15.3
**Invasive UPEC (n = 264)**	**302.7**	**293.4**	**72.9**	**13.7**	**48.4**	**16.9**	**71.6**	**62.3**	**9.4**	**56.4**	**15.6**

*significantly ($P < 0.05$, two-sided Mann-Whitney U test, Bonferroni corrected) increased/decreased compared to invasive UPEC
**significantly ($P < 0.001$, two-sided Mann-Whitney U test, Bonferroni corrected) increased/decreased compared to invasive UPEC

Phylogroup B2: Exact *P* values

Clinical phenotype	VAGs total	VAGs total excl. pap	Iron uptake	Immune evasion and modulation	Secretion system/ Auto-transporter	Toxins	Adhesion and invasion	Adhesion and invasion excl. pap	Bacterio-cins	Flagella	Other
Fecal isolates (n = 76)	1.31E-04	1.26E-03	2.30E-09	1.00E+00	1.00E+00	3.15E-05	7.66E-07	2.41E-03	1.98E-02	1.00E+00	1.00E+00
ABU (n = 65)	1.38E-06	7.49E-05	4.40E-03	6.78E-03	3.12E-04	2.36E-04	3.36E-07	6.15E-04	2.56E-05	4.78E-03	1.59E-01
Cystitis (n = 136)	1.00E+00	1.00E+00	8.75E-06	5.37E-03	1.00E+00	7.97E-02	1.00E+00	5.10E-02	1.00E+00	6.06E-01	8.71E-01
Invasive UPEC (n = 264)	-	-	-	-	-	-	-	-	-	-	-

Supplementary Table 7. Number of virulence-associated genes (VAGs) per isolate by clinical phenotype and phylogroup.

Average number of VAGs per isolate stratified by clinical phenotype and phylogroup. *P* values (two-sided Mann-Whitney U test, Bonferroni corrected) refer to comparisons to invasive UPEC isolates within the same phylogroup. ABU: asymptomatic bacteriuria, UPEC: uropathogenic *E. coli*

Clinical phenotype	A (n = 99)	B1 (n = 63)	B2 (n = 541)	C (n = 25)	D (n = 135)	E or cladeI (n = 10)	F (n = 26)	G (n = 8)
Fecal isolates (n = 185)	218.4	249.6	285.5***	-	281.7***	243.0	293.0	250.0
ABU (n = 113)	233.4	250.8	284.7***	281.2	276.5**	258.0	271.0	248.5
Cystitis (n = 224)	248.8	257.1	299.6	271.0	297.6	258.2	281.5	-
Invasive UPEC (n = 385)	235.8	256.0	302.7	280.5	314.6	269.0	281.3	252.5
Overall (n = 907)	232.6	252.4	297.4	279.1	301.3	256.2	284.6	249.9

Within phylogroup comparisons
**significantly ($P < 0.01$, two-sided Mann-Whitney U test, Bonferroni corrected) decreased compared to invasive UPEC
***significantly ($P < 0.001$, two-sided Mann-Whitney U test, Bonferroni corrected) decreased compared to invasive UPEC

Exact *P* values

Clinical phenotype	A (n = 99)	B1 (n = 63)	B2 (n = 541)	C (n = 25)	D (n = 135)	E or cladeI (n = 10)	F (n = 26)	G (n = 8)
Fecal isolates (n = 185)	1.00E+00	1.00E+00	1.31E-04	-	1.86E-05	1.00E+00	5.22E-01	1.00E+00
ABU (n = 113)	1.00E+00	1.00E+00	1.38E-06	1.00E+00	1.02E-03	1.00E+00	1.00E+00	1.00E+00
Cystitis (n = 224)	5.89E-01	1.00E+00	1.00E+00	6.79E-01	5.51E-02	1.00E+00	1.00E+00	-
Invasive UPEC (n = 385)	-	-	-	-	-	-	-	-

Supplementary Table 8. Average number of virulence-associated genes (VAGs) for *papGII*+ isolates associated with different clinical phenotypes.

The average number of VAGs is shown with 95% percentile confidence intervals (CI) by bootstrap. Differences were not statistically significant (two-sided Mann-Whitney U test, Bonferroni corrected). ABU: asymptomatic bacteriuria, UPEC: uropathogenic *E. coli*

Clinical phenotype	No. isolates	Exact *P* values (vs. invasive UPEC)	Average no. VAGs (95% CI)
Fecal isolates	35	0.50	301.3 (296.2 - 306.2)
ABU	10	0.29	295.0 (283.3 - 306.8)
Cystitis	42	1.00	306.9 (301.4 - 312.5)
Invasive UPEC	246	-	307.6 (305.1 - 310.2)

Supplementary Table 9. Average number of iron uptake systems in isolates of different clinical phenotypes or genotypes by phylogroup.

Numbers are based on the presence of 22 representative genes of iron uptake systems (see Supplementary Table 10) identified using BLASTn. *P* values (two-sided Mann-Whitney U test, Bonferroni corrected) refer to comparisons to invasive UPEC isolates or *papGII*+ isolates within the same phylogroup. ABU: asymptomatic bacteriuria, UPEC: uropathogenic *E. coli*

	All phylogroups	A	B1	B2	C	D	E or cladeI	F	G
Clinical phenotype									
Fecal isolates (n = 185)	12.9***	10.7	9.8	15.1***	-	12.5***	11.0	15.8	13.0
ABU (n = 113)	14.0***	10.8	11.1	15.5***	13.0	12.8**	14.0	14.0	15.0
Cystitis (n = 224)	14.3***	11.6	11.1	15.5***	13.3	13.7	12.3	15.5	-
**Invasive UPEC (n = 385)**	**15.6**	**12.1**	**10.8**	**16.5**	**12.6**	**14.4**	**14.0**	**15.2**	**15.5**
Genotype									
***papGII*+ isolates (n = 333)**	**16.3**	**13.7**	**13.0**	**17.0**	**13.3**	**14.8**	**-**	**15.5**	**15.0**
Other (n = 574)	13.5***	11.0**	10.4	15.1***	12.7	12.6***	12.4	14.8	14.6

**significantly ($P < 0.01$, two-sided Mann-Whitney U test, Bonferroni corrected) decreased compared to invasive UPEC (clinical phenotype) or *papGII*+ isolates (genotype)
***significantly ($P < 0.001$, two-sided Mann-Whitney U test, Bonferroni corrected) decreased compared to invasive UPEC (clinical phenotype) or *papGII*+ isolates (genotype)

Exact *P* values

	All phylogroups	A	B1	B2	C	D	E or cladeI	F	G
Clinical phenotype									
Fecal isolates (n = 185)	2.20E-24	9.04E-02	1.39E-01	1.03E-08	-	4.13E-06	1.00E+00	1.00E+00	6.62E-01
ABU (n = 113)	1.21E-08	6.09E-01	1.00E+00	1.61E-05	1.00E+00	9.93E-03	-	1.00E+00	1.00E+00
Cystitis (n = 224)	2.21E-11	1.00E+00	1.00E+00	2.97E-09	8.51E-01	4.79E-01	1.00E+00	1.00E+00	-
Invasive UPEC (n = 385)	-	-	-	-	-	-	-	-	-
Genotype									
papGII+ isolates (n = 333)	-	-	-	-	-	-	-	-	-
Other (n = 574)	2.26E-63	2.93E-03	8.31E-02	4.10E-49	2.95E-01	3.70E-15	-	3.96E-01	1.00E+00

Supplementary Table 10. Frequency of 22 iron uptake systems by clinical phenotype and phylogroup.

Distribution of representative genes of each iron uptake system identified using BLASTn in (a) the complete dataset and (b) isolates of phylogroup B2. *P* values (two-sided Fisher's exact test, Bonferroni corrected for 22 tests) and odds ratios (OR) with 95% confidence intervals (CI) are shown for genes with significant differences in prevalence rates between non-invasive (asymptomatic bacteriuria (ABU) and cystitis) and invasive uropathogenic *E. coli* (UPEC) isolates. (c) Distribution of representative genes in *E. coli* phylogroups.

a

Gene	Fecal isolates (n = 185)	ABU (n = 113)	Cystitis (n = 224)	Invasive UPEC (n = 385)	*P* (invasive vs. non-invasive UPEC)	Exact *P* (invasive vs. non-invasive UPEC)	OR (95% CI) (invasive vs. non-invasive UPEC)
Siderophore systems							
entA	185 (100%)	112 (99.1%)	224 (100%)	385 (100%)	1.0	1.00E+00	
fyuA	108 (58.4%)	84 (74.3%)	177 (79.0%)	358 (93.0%)	<0.001	4.92E-08	3.9 (2.4 - 6.4)
iroB	46 (24.9%)	38 (33.6%)	112 (50.0%)	183 (47.5%)	1.0	1.00E+00	
iucA	65 (35.1%)	54 (47.8%)	81 (36.2%)	280 (72.7%)	<0.001	1.23E-17	4.0 (2.9 - 5.5)
Other iron uptake systems							
chuA	115 (62.2%)	82 (72.6%)	173 (77.2%)	346 (89.9%)	<0.001	9.59E-06	2.8 (1.9 - 4.4)
cirA	183 (98.9%)	112 (99.1%)	223 (99.6%)	384 (99.7%)	1.0	1.00E+00	
cjrA	45 (24.3%)	24 (21.2%)	55 (24.6%)	143 (37.1%)	0.002	1.60E-03	1.9 (1.4 - 2.7)
efeB	185 (100%)	111 (98.2%)	224 (100%)	385 (100%)	1.0	1.00E+00	
eitA	7 (3.8%)	3 (2.7%)	7 (3.1%)	15 (3.9%)	1.0	1.00E+00	
fbpA	6 (3.2%)	4 (3.5%)	11 (4.9%)	27 (7.0%)	1.0	1.00E+00	
fecA	64 (34.6%)	75 (66.4%)	147 (65.6%)	263 (68.3%)	1.0	1.00E+00	
feoA	185 (100%)	113 (100%)	224 (100%)	385 (100%)	1.0	1.00E+00	
fhuA	111 (60.0%)	44 (38.9%)	102 (45.5%)	123 (31.9%)	0.04	4.41E-02	0.6 (0.4 - 0.8)
fhuE	184 (99.5%)	112 (99.1%)	223 (99.6%)	385 (100%)	1.0	1.00E+00	
fitA	108 (58.4%)	76 (67.3%)	152 (67.9%)	286 (74.3%)	1.0	1.00E+00	
Fiu	183 (98.9%)	113 (100%)	224 (100%)	384 (99.7%)	1.0	1.00E+00	
Hma	55 (29.7%)	55 (48.7%)	115 (51.3%)	225 (58.4%)	0.8	7.89E-01	
Iha	48 (25.9%)	43 (38.1%)	67 (29.9%)	209 (54.3%)	<0.001	1.40E-07	2.4 (1.8 - 3.4)
ireA	29 (15.7%)	14 (12.4%)	34 (15.2%)	128 (33.2%)	<0.001	5.39E-08	3.0 (2.0 - 4.4)
mntH	185 (100%)	113 (100%)	224 (100%)	385 (100%)	1.0	1.00E+00	
sitA	109 (58.9%)	89 (78.8%)	174 (77.7%)	353 (91.7%)	<0.001	6.27E-06	3.1 (2.0 - 5.0)
zupT	185 (100%)	113 (100%)	224 (100%)	384 (99.7%)	1.0	1.00E+00	

b

Phylogroup B2

Gene	Fecal isolates (n = 76)	ABU (n = 65)	Cystitis (n = 136)	Invasive UPEC (n = 264)	*P* (invasive vs. non-invasive UPEC)	Exact *P* (invasive vs. non-invasive UPEC)	OR (95% CI) (invasive vs. non-invasive UPEC)
Siderophore systems							
entA	76 (100%)	65 (100%)	136 (100%)	264 (100%)	1.0	1.00E+00	
fyuA	72 (94.7%)	64 (98.5%)	132 (97.1%)	263 (99.6%)	1.0	1.00E+00	
iroB	41 (53.9%)	27 (41.5%)	103 (75.7%)	165 (62.5%)	1.0	1.00E+00	
iucA	38 (50.0%)	36 (55.4%)	50 (36.8%)	187 (70.8%)	<0.001	3.52E-08	3.2 (2.2 - 4.9)
Other iron uptake systems							
chuA	75 (98.7%)	64 (98.5%)	136 (100%)	263 (99.6%)	1.0	1.00E+00	
cirA	75 (98.7%)	65 (100%)	135 (99.3%)	264 (100%)	1.0	1.00E+00	
cjrA	29 (38.1%)	19 (29.2%)	34 (25.0%)	105 (39.8%)	0.1	6.56E-02	
efeB	76 (100%)	64 (98.5%)	136 (100%)	264 (100%)	1.0	1.00E+00	
eitA	4 (5.2%)	1 (1.5%)	5 (3.7%)	14 (5.3%)	1.0	1.00E+00	
fbpA	1 (1.3%)	1 (1.5%)	4 (2.9%)	22 (8.3%)	0.2	1.86E-01	
fecA	18 (23.7%)	41 (63.1%)	76 (55.9%)	157 (59.5%)	1.0	1.00E+00	
feoA	76 (100%)	65 (100%)	136 (100%)	264 (100%)	1.0	1.00E+00	
fhuA	31 (40.8%)	14 (21.5%)	45 (33.1%)	90 (34.1%)	1.0	1.00E+00	
fhuE	76 (100%)	64 (98.5%)	136 (100%)	264 (100%)	1.0	1.00E+00	
fitA	76 (100%)	64 (98.5%)	133 (97.8%)	263 (99.6%)	1.0	1.00E+00	
fiu	74 (97.4%)	65 (100%)	136 (100%)	263 (99.6%)	1.0	1.00E+00	
hma	48 (63.2%)	53 (81.5%)	105 (77.2%)	209 (79.2%)	1.0	1.00E+00	
iha	23 (30.3%)	35 (53.8%)	36 (26.5%)	137 (51.9%)	0.01	1.06E-02	2.0 (1.3 - 2.9)
ireA	19 (25.0%)	5 (7.7%)	30 (22.1%)	117 (44.3%)	<0.001	1.04E-08	3.8 (2.4 - 6)
mntH	76 (100%)	65 (100%)	136 (100%)	264 (100%)	1.0	1.00E+00	
sitA	71 (93.4%)	63 (96.9%)	126 (92.6%)	252 (95.5%)	1.0	1.00E+00	
zupT	76 (100%)	65 (100%)	136 (100%)	263 (99.6%)	1.0	1.00E+00	

c

Gene	A (n = 99)	B1 (n = 63)	B2 (n = 541)	C (n = 25)	D (n = 135)	E or cladeI (n = 10)	F (n = 26)	G (n = 8)
Siderophore systems								
entA	99 (100%)	63 (100%)	541 (100%)	25 (100%)	134 (99.3%)	10 (100%)	26 (100%)	8 (100%)
fyuA	36 (36.4%)	13 (20.6%)	531 (98.2%)	23 (92.0%)	92 (68.1%)	1 (10.0%)	25 (96.2%)	6 (75.0%)
iroB	4 (4.0%)	9 (14.3%)	336 (62.1%)	17 (68.0%)	8 (5.9%)	0 (0.0%)	1 (3.8%)	4 (50.0%)
iucA	24 (24.2%)	11 (17.5%)	311 (57.5%)	19 (76.0%)	85 (63.0%)	1 (10.0%)	24 (92.3%)	5 (62.5%)
Other iron uptake systems								
chuA	0 (0.0%)	0 (0.0%)	538 (99.4%)	0 (0.0%)	135 (100%)	9 (90.0%)	26 (100%)	8 (100%)
cirA	99 (100%)	61 (96.8%)	539 (99.6%)	25 (100%)	134 (99.3%)	10 (100%)	26 (100%)	8 (100%)
cjrA	16 (16.2%)	1 (1.6%)	187 (34.6%)	0 (0.0%)	51 (37.8%)	0 (0.0%)	11 (42.3%)	1 (12.5%)
efeB	99 (100%)	63 (100%)	540 (99.8%)	25 (100%)	135 (100%)	9 (90.0%)	26 (100%)	8 (100%)
eitA	2 (2.0%)	0 (0.0%)	24 (4.4%)	1 (4.0%)	4 (3.0%)	0 (0.0%)	0 (0%)	1 (12.5%)
fbpA	3 (3.0%)	0 (0.0%)	28 (5.2%)	1 (4.0%)	3 (2.2%)	10 (100%)	2 (7.7%)	1 (12.5%)
fecA	70 (70.7%)	27 (42.9%)	292 (54.0%)	25 (100%)	103 (76.3%)	7 (70.0%)	20 (76.9%)	5 (62.5%)
feoA	99 (100%)	63 (100%)	541 (100%)	25 (100%)	135 (100%)	10 (100%)	26 (100%)	8 (100%)
fhuA	95 (96.0%)	62 (98.4%)	180 (33.3%)	0 (0.0%)	35 (25.9%)	6 (60.0%)	2 (7.7%)	0 (0%)
fhuE	98 (99.0%)	63 (100%)	540 (99.8%)	25 (100%)	134 (99.3%)	10 (100%)	26 (100%)	8 (100%)
fitA	3 (3.0%)	0 (0.0%)	536 (99.1%)	0 (0.0%)	57 (42.2%)	3 (30.0%)	15 (57.7%)	8 (100%)
fiu	99 (100%)	63 (100%)	538 (99.4%)	25 (100%)	135 (100%)	10 (100%)	26 (100%)	8 (100%)
hma	4 (4.0%)	3 (4.8%)	415 (76.7%)	0 (0.0%)	14 (10.4%)	0 (0.0%)	14 (53.8%)	0 (0%)
iha	25 (25.3%)	7 (11.1%)	231 (42.7%)	2 (8.0%)	77 (57.0%)	2 (20.0%)	21 (80.8%)	2 (25.0%)
ireA	3 (3.0%)	7 (11.1%)	171 (31.6%)	7 (28.0%)	2 (1.5%)	2 (20.0%)	6 (23.1%)	7 (87.5%)
mntH	99 (100%)	63 (100%)	541 (100%)	25 (100%)	135 (100%)	10 (100%)	26 (100%)	8 (100%)
sitA	29 (29.3%)	17 (27.0%)	512 (94.6%)	24 (96.0%)	110 (81.5%)	4 (40.0%)	24 (92.3%)	5 (62.5%)
zupT	99 (100%)	63 (100%)	540 (99.8%)	25 (100%)	135 (100%)	10 (100%)	26 (100%)	8 (100%)

Supplementary Table 11. Description of the source collections of *E. coli* isolates included in the CC131 dataset.

Genome assemblies of 1,017 CC131 isolates were pooled with genome assemblies of 59 CC131 isolates from the main dataset. Details of all 1,076 isolates are provided in Supplementary Data 10. UTI: urinary tract infection, UPEC: uropathogenic *E. coli*, ESBL: extended spectrum beta-lactamase

Collection	No. isolates	Year of collection	Population	Clinical phenotype	Clinical phenotype (grouped)	Reference
Enterobase	799	na	unknown	na	na	Zhou et al.[33]
NCBI near-complete genomes	48	na	various	na	non-invasive UPEC (n = 1), na (n = 48)	
Petty_2014	52	na	various	various	invasive UPEC (n = 9), non-invasive UPEC (n = 14), na (n = 30)	Petty et al.[34]
Birgy_2019	92	2014 - 2017	children with febrile UTI caused by ESBL-producing *E. coli*	febrile UTI	invasive UPEC	Birgy et al.[35]
Syre_2020	16	2013 - 2016	women with catheter-associated cystitis caused by ESBL-producing *E. coli*	cystitis	non-invasive UPEC	Syre et al.[36]
Goswami_2018	10	2013 - 2015	general population	urinary-source bacteremia	invasive UPEC	Goswami et al.[37]

Supplementary Table 12. Resolved *papGII*-containing pathogenicity islands (*papGII*+ PAIs) identified in complete or near-complete assemblies of CC131 isolates.

Additional *papGII*+ PAI types from CC131 isolates with low-quality genome assemblies as identified by read mapping are provided in Supplementary Data 12.

Isolate	*papGII*+ PAI type	CC131 subclade
AR_0378	Type III	C2
B1017-PB	Type III	C2
B1033-PB	Type III	C2
B1131-PB	Type III	C2
B1316-PB	Type III	C2
B1320-PB	Type III	C2
B1323-PB	Type III	C2
B1370-PB	Type III	C2
C0014-PB	Type III	C2
C0107-PB	Type III	C2
C0134-PB	Type III	C2
EC_2_0	Type IV	C2
EC_4_0	Type III	C2
Ecol_656	Type III	C2
Ecol_AZ146	Type III	C2
FDAARGOS_142	Type III	C2
O25b_H4	Type III	C2
S65EC	Type III	C2
TO217	Type III	C2
US02	Type III	C2
U-NHP-94	Type III	C1

References

1. Bonat, G. *et al.* European Association of Urology Guidelines on Urological Infections. *EAU Guidel.* 1–66 (2018).
2. Sandberg, T. *et al.* Ciprofloxacin for 7 days versus 14 days in women with acute pyelonephritis: a randomised, open-label and double-blind, placebo-controlled, non-inferiority trial. *Lancet* **380**, 484–490 (2012).
3. Kim, S.-H. *et al.* Inappropriate empirical antibiotic therapy does not adversely affect the clinical outcomes of patients with acute pyelonephritis caused by extended-spectrum β-lactamase-producing Enterobacteriales. *Eur. J. Clin. Microbiol. Infect. Dis.* **38**, 937–944 (2019).
4. Hyun, M., Lee, J. Y., Kim, H. ah & Ryu, S. Y. Comparison of *Escherichia coli* and *Klebsiella pneumoniae* Acute Pyelonephritis in Korean Patients. *Infect. Chemother.* **51**, 130 (2019).
5. Riley, L. W. Pandemic lineages of extraintestinal pathogenic *Escherichia coli*. *Clin. Microbiol. Infect.* **20**, 380–390 (2014).
6. Galardini, M. *et al.* Major role of the high-pathogenicity island (HPI) in the intrinsic extra-intestinal virulence of *Escherichia coli* revealed by a genome-wide association study. *'Preprint at bioRxiv'* (2019) doi:https://doi.org/10.1101/712034.
7. Spurbeck, R. R. *et al. Escherichia coli* Isolates That Carry *vat*, *fyuA*, *chuA*, and *yfcV* Efficiently Colonize the Urinary Tract. *Infect. Immun.* **80**, 4115–4122 (2012).
8. Original S code by Richard A. Becker, Allan R. Wilks. R version by Ray Brownrigg. Enhancements by Thomas P Minka and Alex Deckmyn. maps: Draw Geographical Maps. R package version 3.3.0. https://CRAN.R-project.org/package=maps. (2018).
9. Edgar, R. C. MUSCLE: A multiple sequence alignment method with reduced time and space complexity. *BMC Bioinformatics* **5**, 1–19 (2004).
10. Sullivan, M. J., Petty, N. K. & Beatson, S. A. Easyfig: A genome comparison visualizer. *Bioinformatics* **27**, 1009–1010 (2011).
11. Revell, L. J. phytools: an R package for phylogenetic comparative biology (and other things). *Methods Ecol. Evol.* **3**, 217–223 (2012).
12. Letunic, I. & Bork, P. Interactive Tree Of Life (iTOL) v4: recent updates and new developments. *Nucleic Acids Res.* **47**, W256–W259 (2019).
13. Lalioui, L., Jouve, M., Gounon, P. & Le Bouguenec, C. Molecular Cloning and Characterization of the *afa-7* and *afa-8 Gene* Clusters Encoding Afimbrial Adhesins in *Escherichia coli* Strains Associated with Diarrhea or Septicemia in Calves. *Infect. Immun.* **67**, 5048–5059 (1999).
14. Waterhouse, A. M., Procter, J. B., Martin, D. M. A., Clamp, M. & Barton, G. J. Jalview Version 2 - a multiple sequence alignment editor and analysis workbench. *Bioinformatics* **25**, 1189–1191 (2009).
15. Johnson, J. R. *et al.* Host Characteristics and Bacterial Traits Predict Experimental Virulence for *Escherichia coli* Bloodstream Isolates From Patients With Urosepsis. *Open Forum Infect. Dis.* **2**, ofu121 (2015).
16. Johnson, J. R. & Stell, A. L. Extended Virulence Genotypes of *Escherichia coli* Strains from Patients with Urosepsis in Relation to Phylogeny and Host Compromise. *J. Infect. Dis.* **181**, 261–272 (2000).
17. Schreiber, H. L. *et al.* Bacterial virulence phenotypes of *Escherichia coli* and host susceptibility determine risk for urinary tract infections. *Sci. Transl. Med.* **9**, eaaf1283 (2017).
18. Ben Zakour, N. L. *et al.* Sequential Acquisition of Virulence and Fluoroquinolone Resistance Has Shaped the Evolution of *Escherichia coli* ST131. *MBio* **7**, 1–11 (2016).

19. Stork, C. *et al.* Characterization of Asymptomatic Bacteriuria *Escherichia coli* Isolates in Search of Alternative Strains for Efficient Bacterial Interference against Uropathogens. *Front. Microbiol.* **9**, 1–18 (2018).
20. Coussement, J. *et al.* Host and microbial factors in kidney transplant recipients with *Escherichia coli* acute pyelonephritis or asymptomatic bacteriuria: a prospective study using whole-genome sequencing. *Nephrol. Dial. Transplant.* **34**, 878–885 (2019).
21. Drekonja, D. M., Kuskowski, M. A., Anway, R., Johnston, B. D. & Johnson, J. R. The niche for *Escherichia coli* sequence type 131 among veterans: Urinary tract abnormalities and long-term care facilities. *Open Forum Infect. Dis.* **3**, 1–6 (2016).
22. Skjøt-Rasmussen, L. *et al.* Persisting clones of *Escherichia coli* isolates from recurrent urinary tract infection in men and women. *J. Med. Microbiol.* **60**, 550–554 (2011).
23. Nielsen, K. L. *et al.* Whole-genome comparison of urinary pathogenic *Escherichia coli* and faecal isolates of UTI patients and healthy controls. *Int. J. Med. Microbiol.* **307**, 497–507 (2017).
24. Johnson, J. R. *et al.* Accessory Traits and Phylogenetic Background Predict *Escherichia coli* Extraintestinal Virulence Better Than Does Ecological Source. *J. Infect. Dis.* **219**, 121–132 (2018).
25. Sannes, M. R., Kuskowski, M. A. & Johnson, J. R. Antimicrobial resistance of *Escherichia coli* strains isolated from urine of women with cystitis or pyelonephritis and feces of dogs and healthy humans. *J. Am. Vet. Med. Assoc.* **225**, 368–373 (2004).
26. Ejrnæs, K. *et al.* Characteristics of *Escherichia coli* causing persistence or relapse of urinary tract infections: Phylogenetic groups, virulence factors and biofilm formation. *Virulence* **2**, 528–537 (2011).
27. Czaja, C. A. C. *et al.* Prospective Cohort Study of Microbial and Inflammatory Events Immediately Preceding *Escherichia coli* Recurrent Urinary Tract Infection in Women. *J. Infect. Dis.* **200**, 528–536 (2009).
28. Sannes, M. R., Kuskowski, M. A. & Johnson, J. R. Geographical distribution of antimicrobial resistance among *Escherichia coli* causing acute uncomplicated pyelonephritis in the United States. *FEMS Immunol. Med. Microbiol.* **42**, 213–218 (2004).
29. Talan, D. A. *et al.* Comparison of Ciprofloxacin (7 Days) and Trimethoprim-Sulfamethoxazole (14 Days) for Acute Uncomplicated Pyelonephritis in Women. *JAMA* **283**, 1583 (2000).
30. Skjøt-Rasmussen, L., Ejrnæs, K., Lundgren, B., Hammerum, A. M. & Frimodt-Møller, N. Virulence factors and phylogenetic grouping of *Escherichia coli* isolates from patients with bacteraemia of urinary tract origin relate to sex and hospital- vs. community-acquired origin. *Int. J. Med. Microbiol.* **302**, 129–134 (2012).
31. Dale, A. P. *et al.* Genomes of *Escherichia coli* bacteraemia isolates originating from urinary tract foci contain more virulence-associated genes than those from non-urinary foci and neutropaenic hosts. *J. Infect.* **77**, 534–543 (2018).
32. Sannes, M. R., Kuskowski, M. A., Owens, K., Gajewski, A. & Johnson, J. R. Virulence Factor Profiles and Phylogenetic Background of *Escherichia coli* Isolates from Veterans with Bacteremia and Uninfected Control Subjects. *J. Infect. Dis.* **190**, 2121–2128 (2004).
33. Zhou, Z., Alikhan, N., Mohamed, K., Fan, Y. & Achtman, M. The EnteroBase user's guide, with case studies on *Salmonella* transmissions, *Yersinia pestis* phylogeny, and *Escherichia* core genomic diversity. *Genome Res.* **30**, 138–152 (2020).
34. Petty, N. K. *et al.* Global dissemination of a multidrug resistant *Escherichia coli* clone. *Proc. Natl. Acad. Sci.* **111**, 5694–5699 (2014).
35. Birgy, A. *et al.* Diversity and trends in population structure of ESBL-producing

Enterobacteriaceae in febrile urinary tract infections in children in France from 2014 to 2017. *J. Antimicrob. Chemother.* 96–105 (2019) doi:10.1093/jac/dkz423.
36. Syre, H. *et al.* Microbial risk factors for treatment failure of pivmecillinam in community-acquired urinary tract infections caused by ESBL-producing *Escherichia coli*. *APMIS* apm.13013 (2020) doi:10.1111/apm.13013.
37. Goswami, C. *et al.* Genetic analysis of invasive *Escherichia coli* in Scotland reveals determinants of healthcare-associated versus community-acquired infections. *Microb. Genomics* **4**, 1–15 (2018).
