# Supplementary material for: Horizontally acquired papGII-containing pathogenicity islands underlie the emergence of invasive uropathogenic Escherichia coli lineages
Source: Nat Commun. 2020 Nov 24;11:5968. doi: 10.1038/s41467-020-19714-9 (PMC7686366; doi:10.1038/s41467-020-19714-9)
Supplement: Supplementary file 3 — Description of Additional Supplementary Files [file 41467_2020_19714_MOESM3_ESM.pdf]

**Description of Additional Supplementary Files**

File Name: Supplementary Data 1
Description: **Characteristics of 907 bacterial isolates included in the main dataset.** Each isolate is annotated with the accession number, assembly metrics, clinical metadata, and results of genotypic analyses.

File Name: Supplementary Data 2
Description: **Bayesian Analysis of Population Structure (BAPS) group levels of the 907 isolates and associations with invasive uropathogenic E. coli (UPEC) isolates**. Clustering was performed using fastbaps. P values are provided for associations with invasive UPEC isolates (two-tailed fisher's exact test).

File Name: Supplementary Data 3
Description: **Results of genome-wide association studies based on linear mixed-models (DBGWAS).** DBGWAS results for significant associations of invasive vs. non-invasive UPEC isolates. The table provides counts, statistical data, and nucleotide sequences for nodes of 27 components (compacted De Brujin graphs) containing nodes with $q < 0.05$.

File Name: Supplementary Data 4
Description: **Clusters of orthologues genes (COGs) associated with invasive uropathogenic E. coli (UPEC) isolates vs. non-invasive UPEC isolates in a pan-genome wide association analysis.** COGs with raw P values (two tailed fisher's exact test) below the simulation inferred significance threshold of P = 1.42-18 are shown, as well as COGs with Bonferroni adjusted P values below P = 0.05.

File Name: Supplementary Data 5
Description: **Characteristics of 24 representative invasive uropathogenic E. coli (UPEC) isolates sequenced using PacBio long-read sequencing technology.** Accession numbers, assembly metrics, phylogenetic affiliation, and key virulence elements are annotated.

File Name: Supplementary Data 6
Description: **Contig-wise details on the 24 assemblies obtained from long-read sequencing**. Assembly methods, prediction of plasmid- or chromosome-derived sequences, and identified plasmid replicons are given.

File Name: Supplementary Data 7
Description: **Characteristics of 333 papGII+ E. coli isolates and associated pathogenicity islands.** Phylogenetic affiliation, identified iuc variants, papGII+ PAI types, and insertion sites are annotated.

File Name: Supplementary Data 8
Description: **Virulence-associated gene profiles of the 907 E. coli isolates.** Results of a BLASTn-based screening using the EcVGDB database. Results may deviate in some cases from read-mapping inferred results shown in Supplementary Data 9 due to fragmented genome assemblies from short-read data.

File Name: Supplementary Data 9
Description: **Profile of papG and afa-family genes of the 907 E. coli isolates.** Data are based on read-mapping when read data available (Supplementary Table 1), otherwise identified using BLASTn.

File Name: Supplementary Data 10
Description: **Characteristics of 1,076 isolates included in the CC131 dataset.** Each isolate is annotated with the accession number and results of genotypic analyses.

File Name: Supplementary Data 11
Description: **Read-mapping based identification of papGII+ pathogenicity islands for papGII+ isolates of the main dataset.** Identified papGII+ PAIs hits and srst2 read-mapping metrics are shown.

File Name: Supplementary Data 12
Description: **Read-mapping based identification of papGII+ pathogenicity islands for papGII+ isolates of the CC131 dataset.** Analyses performed for papGII+ isolates with available read-data were included. Identified papGII+ PAIs hits and srst2 read-mapping metrics are shown.
