# Supplementary material for: Horizontally acquired papGII-containing pathogenicity islands underlie the emergence of invasive uropathogenic Escherichia coli lineages
Source: Nat Commun. 2020 Nov 24;11:5968. doi: 10.1038/s41467-020-19714-9 (PMC7686366; doi:10.1038/s41467-020-19714-9)
Supplement: Supplementary file 5 — Reporting Summary [file 41467_2020_19714_MOESM5_ESM.pdf]

nature research

Corresponding author(s): Sandra Van Puyvelde, Michael Biggel

Last updated by author(s): 21/12/20

Reporting Summary

Nature Research wishes to improve the reproducibility of the work that we publish. This form provides structure for consistency and transparency in reporting. For further information on Nature Research policies, see our Editorial Policies and the Editorial Policy Checklist.

Statistics

For all statistical analyses, confirm that the following items are present in the figure legend, table legend, main text, or Methods section.

n/a	Confirmed	
[ ]	[x]	The exact sample size (n) for each experimental group/condition, given as a discrete number and unit of measurement
[x]	[ ]	A statement on whether measurements were taken from distinct samples or whether the same sample was measured repeatedly
[ ]	[x]	The statistical test(s) used AND whether they are one- or two-sided<br>*Only common tests should be described solely by name; describe more complex techniques in the Methods section.*
[x]	[ ]	A description of all covariates tested
[ ]	[x]	A description of any assumptions or corrections, such as tests of normality and adjustment for multiple comparisons
[ ]	[x]	A full description of the statistical parameters including central tendency (e.g. means) or other basic estimates (e.g. regression coefficient) AND variation (e.g. standard deviation) or associated estimates of uncertainty (e.g. confidence intervals)
[ ]	[x]	For null hypothesis testing, the test statistic (e.g. F, t, r) with confidence intervals, effect sizes, degrees of freedom and P value noted<br>*Give P values as exact values whenever suitable.*
[x]	[ ]	For Bayesian analysis, information on the choice of priors and Markov chain Monte Carlo settings
[x]	[ ]	For hierarchical and complex designs, identification of the appropriate level for tests and full reporting of outcomes
[x]	[ ]	Estimates of effect sizes (e.g. Cohen's d, Pearson's r), indicating how they were calculated

Our web collection on statistics for biologists contains articles on many of the points above.

Software and code

Policy information about availability of computer code

Data collection	No software was used for data collection.
Data analysis	Publicly available software:<br>TrimGalore v0.4.4<br>Spades v3.13.0<br>HGAP 4<br>Unicycler v0.4.8<br>Jalview 2.11<br>MLPlasmids 1.0.0<br>ABRicate v0.9.3<br>CLC Sequence Viewer v8.0<br>PyANI v0.2.9<br>Clustal2.1<br>Mega-X v10.0.5<br>Muscle v3.8.31<br>EasyFig 2.2.3<br>Prokka v1.13.3<br>mashtree v1.12<br>R v3.5.3<br>phytools v0.6-60<br>R package maps 3.3.0<br>Pointfinder (release 3.1.0)

Parsnp v1.2
PHASTER (no version tag available; https://phaster.ca accessed in October 2019)
RaXML v8.2.12
ClonalFrameML v1.12
fastbaps v1.0.0
RAMI (no version tag available; http://130.235.244.92/rami.html accessed in October 2019)
iTOL 5
Inkscape 0.92
Roary v3.12.0
treeWAS v1.0
CMplot v.3.3.3
IslandViewer 4
DBGWAS v0.5.4
ClermonTyping v1.3
mlst v2.16.1
srst2 v0.2.0
FimTyper v1.1

For manuscripts utilizing custom algorithms or software that are central to the research but not yet described in published literature, software must be made available to editors and reviewers. We strongly encourage code deposition in a community repository (e.g. GitHub). See the Nature Research guidelines for submitting code & software for further information.

Data

Policy information about availability of data

All manuscripts must include a data availability statement. This statement should provide the following information, where applicable:

- Accession codes, unique identifiers, or web links for publicly available datasets
- A list of figures that have associated raw data
- A description of any restrictions on data availability

1. Availability of data generated as part of this study:

Illumina and PacBio reads generated in this study are available at the NCBI Sequence Read Archive (SRA) under BioProject no. PRJNA592372. Individual accession numbers are provided in Supplementary Data 1.

Complete or draft genome assemblies have been submitted to NCBI GenBank (BioProject no. PRJNA592372). Individual accession numbers are provided in Supplementary Data 1.

Sequences of papGII+ PAIs and a curated E. coli virulence gene database (EcVGDB) are provided at https://github.com/MBiggel/UPEC_study (doi:10.5281/zenodo.4079473).

An interactive version of the core genome phylogeny of the 907 E. coli isolates is accessible at https://microreact.org/project/O4QAYAJWw.

All other relevant data are available from the corresponding authors.

2. Public data utilized in this study include genomic data (accession numbers provided in Supplementary Data 1 and 10), the databases plasmidfinder, resfinder, EcOH, ecoli_vf, ecoli_VF_collection, and the EnteroBase ST/CC scheme.

Field-specific reporting

Please select the one below that is the best fit for your research. If you are not sure, read the appropriate sections before making your selection.

☐ Life sciences ☐ Behavioural & social sciences ☒ Ecological, evolutionary & environmental sciences

For a reference copy of the document with all sections, see nature.com/documents/nr-reporting-summary-flat.pdf

Ecological, evolutionary & environmental sciences study design

All studies must disclose on these points even when the disclosure is negative.

Study description	Descriptive study of Escherichia coli isolates obtained from urine, blood, or feces from healthy participants or patients with urinary tract infections.
Research sample	1. Main dataset:<br>- research sample: genomic data of 907 E.coli isolates, comprising 19 reference strains and 888 isolates from 16 collections<br>- sample choice: aims were to investigate genotype-phenotype associations and the population structure of invasive vs. non-invasive UPEC isolates using a balanced dataset. All available and relevant public genomes with well annotated clinical metadata were included and supplemented with genomic data of 151 isolates collected as part of this study or shared by collaborators. The final dataset comprised 385 invasive and 337 non-invasive UPEC isolates from spatiotemporally diverse collections. 185 fecal E.coli isolates

	that were not associated with infection were included for phylogenomic context.<br>- 24 representative isolates (Supplementary Data 5) were additionally whole-genome sequenced using PacBio long-read sequencing to investigate the genetic context of key virulence determinants. The genetic context could usually not be resolved from short-read sequencing data alone.<br>- sample source, accession numbers, and metadata (host age, gender, clinical phenotype) of collections are described in Supplementary Table 1 and Supplementary Data 1<br>2. CC131 dataset<br>- research sample: genomic data of 1,076 CC131 E.coli isolates, comprising 59 genome assemblies from the main dataset pooled with 1,017 publicly available genome assemblies<br>- sample choice: genomic data of 192 CC131 with available metadata on UPEC phenotypes (invasive, non-invasive) were identified and included to investigate genotype-phenotype associations; to investigate the emergence of papGII-containing sublineages, additional public genomic data were added to the analyses<br>- sample source, accession numbers, and metadata (clinical phenotype) of collections are described in Supplementary Table 11 and Supplementary Data 10
Sampling strategy	Literature was searched for collections of genomic data of E.coli associated with asymptomatic bacteriuria, cystitis, pyelonephritis, urinary-source bacteremia, or non-disease associated fecal isolates. Identified collections were included when clinical metadata was provided. To obtained a balanced dataset of invasive vs. non-invasive UPEC isolates, additional E.coli isolates were collected and sequenced as part of this study and pooled with the public dataset. The final sample size was sufficient for performing genome-wide association studies with corrections for multiple testing and for robust population structure analyses.
Data collection	1. Data collection of the publicly available collections is described in the respective studies (Supplementary Tables 1 and 11). Sequencing technologies and source studies of isolate collections from the main dataset are:<br>dsABU - Illumina MiSeq - Stork et al. 2018<br>RT_ABU - Illumina MiSeq - Coussement et al. 2019<br>MVAST_ABU Illumina MiSeq - Drekonja et al. 2016<br>Koege_cys Illumina HiSeq 2000 - Skjøt-Rasmussen et al. 2011<br>KTE_cys - Illumina HiSeq 2000 - Nielsen et al. 2017<br>PUTI_cys - Illumina Genome Analyzer IIx - Sannes et al. 2004<br>UMEA_cys - Illumina HiSeq 2000 - Ejrnæs et al. 2011<br>Rec_cys - Illumina HiSeq 2000 - Czaja et al. 2009<br>MC_pye - Illumina MiSeq and Genome Analyzer IIx - Sannes et al. 2004, Talan et al. 2000<br>HVH_urb - Illumina HiSeq 2000 - Skjøt-Rasmussen et al. 2012<br>UHS_urb - Illumina HiSeq 2000 - Dale et al.<br>BUTI_uro - Illumina Genome Analyzer IIx - Johnson et al. 2018<br>KTE_fec - Illumina HiSeq 2000 - Nielsen et al. 2017<br>MN_fec - Illumina HiSeq 2000 - Sannes et al. 2004<br><br>2. Isolates from two collections were collected and sequenced as part of this study:<br>2.1 LtABU collection: Urine samples were collected at consecutive time points and processed in the Lab of Medical Microbiology (LMM), University of Antwerp. Isolates causing bacteriuria as identified per quantitative urine culture were subjected to MALDI TOF analyses. Clinical data of donors was recorded and screened for signs or symptoms of urinary tract infections. Obtained long-term asymptomatic bacteriuria E.coli isolates were stored and sequenced using Illumina MiSeq at the LMM. Selected isolates were additionally sequenced using the PacBio Sequel system (Supplementary Data 5).<br>2.2 UZA_uro collection: Isolate databases of the University Hospital Antwerp were screened for bloodstream isolates obtained from patients with concurrent positive urine cultures. Duplicates from identified isolates were stored and sequenced using Illumina MiSeq at the LMM. Selected isolates were additionally sequenced using the PacBio Sequel system (Supplementary Data 5).<br><br>3. Isolates from two additional collections (MVAST_ABU, MC_pye) were sequenced as part of this study (Illumina MiSeq and selected isolates with PacBio Sequel, see Supplementary Data 5), but were collected during previously described studies (see Supplementary Table 1).
Timing and spatial scale	1. Isolates from public genomic data or obtained during previous studies were collected between 1981 and 2017 (Supplementary Tables 1 and 11). Included collections and isolate collection intervals are:<br><br>LtABU 2017 - 2018<br>dsABU 2010 - 2012<br>RT_ABU 2012 - 2015<br>MVAST_ABU 2010 - 2011<br>Koege_cys 2005 - 2006<br>KTE_cys 2009 - 2010<br>PUTI_cys 1999 - 2000<br>UMEA_cys 1995 - 1997<br>Rec_cys 2003 - 2006<br>MC_pye 1994 - 1997<br>HVH_urb 2003 - 2005<br>UHS_urb 2015 - 2016<br>BUTI_uro 1981 - 1985<br>UZA_uro 2015 - 2017<br>KTE_fec 2009 - 2010<br>MN_fec 1996 - 2000

	Enterobase (data not available)<br>NCBI near-complete genomes (data not available)<br>Petty_2014 (data not available)<br>Birgy_2019 2014 - 2017<br>Syre_2020 2013 - 2016<br>Goswami_2018 2013 - 2015<br><br>Criteria for the inclusion of UPEC isolate collections were the availability of associated metadata on clinical syndromes and/or medical diagnosis, i.e., asymptomatic bacteriuria (ABU), cystitis, pyelonephritis, urinary-source bacteremia, or urosepsis. Identified collections were included irrespective of their isolation time.
Data exclusions	KTE_fec isolate collection: assemblies of 40 fecal isolates matching urinary isolates from the same patient (based on whole-genome sequencing) were excluded (i.e., duplicates)<br>MN_fec, PUTI_cys, MC_pye: sequencing of these isolates was originally performed based on presence and absence of genotypic features as identified by PCR (ratio features present:absent approximately 1:1). To account for this sampling bias, 35 randomly selected genome assemblies were excluded to reflect the unbiased presence/absence ratio of the original collection.
Reproducibility	Multiple invasiveness-associated UPEC and multiple non-invasiveness associated UPEC isolate collections were included to ensure reproducibility across a broad spatiotemporal origin.<br><br>Main findings of this study were confirmed in an independent dataset of ST131 isolates.<br><br>Two alternative GWAS approaches were applied: a pan-genome based approach (roary + treeWAS) and an alignment free approach based on De Brujin graphs (DBGWAS).<br><br>Bootstrapping was used to verify the robustness of phylogenetic trees.
Randomization	Randomization was not applicable to this study. Isolates from various collections were included, most originating from observational studies.
Blinding	Blinding was not applicable to this study. The population cohort was mostly defined by previous studies. No intervention was conducted among the cohort investigated in this study.

Did the study involve field work? ☐ Yes ☒ No

Reporting for specific materials, systems and methods

We require information from authors about some types of materials, experimental systems and methods used in many studies. Here, indicate whether each material, system or method listed is relevant to your study. If you are not sure if a list item applies to your research, read the appropriate section before selecting a response.

Materials & experimental systems

n/a	Involved in the study
☒	☐ Antibodies
☒	☐ Eukaryotic cell lines
☒	☐ Palaeontology and archaeology
☒	☐ Animals and other organisms
☐	☒ Human research participants
☒	☐ Clinical data
☒	☐ Dual use research of concern

Methods

n/a	Involved in the study
☒	☐ ChIP-seq
☒	☐ Flow cytometry
☒	☐ MRI-based neuroimaging

Human research participants

Policy information about studies involving human research participants

Population characteristics	1. For host population characteristics of previously described isolate collections see the respective studies (Supplementary Tables 1 and 11). Relevant host data, including diagnosis, age, and gender, are summarized in Supplementary Table 1 and Supplementary Data 1.<br>2. Isolates collected as part of this study:<br>2.1 LtABU collection: The study population comprised approximately 330 residents from two long-term care facilities (OCMW Destelbergen and WZC Immaculata Edegem, Belgium). Host data was obtained from 43 participants with long-term ABU. Median age was 86 years (range 70 - 97); 41/42 participants were female (Supplementary Table 1 and Supplementary Data 1).<br>2.2 UZA_uro collection: Median age of the 30 urosepsis patients was 75 (0 - 92). 25/30 patients were female (Supplementary Table 1 and Supplementary Data 1).
Recruitment	The main dataset of this study consisted of genomic data from isolates of 16 collections. The 16 source studies varied in their cohort, inclusion criteria, and isolation time span. Biases potentially impacting the results include<br>- the inclusion of immunocompromised patients or patients with co-morbidities, who are known to be more likely to be infected by less virulent pathogens<br>- collections could not always be time-matched with control collections; results may have been affected by a changing E.coli population structure over time<br><br>1. For recruitment of participants of previously described isolate collections, see the respective studies (Supplementary Tables 1 and 11).<br>2. Isolates collected as part of this study:<br>2.1 LtABU collection: residents from two long-term care facilities were eligible. Residents who were catheterized, had a UTI, or received systemic antibiotic treatment were excluded.<br>2.2 UZA_uro collection: retrospective analyses; recruitment is not applicable.
Ethics oversight	Ethics committee UZA (Commissie voor Medische Ethiek, Universitair Ziekenhuis Antwerpen/Universiteit Antwerpen)

Note that full information on the approval of the study protocol must also be provided in the manuscript.
